# Interplay of pericentromeric genome organization and chromatin landscape regulates the expression of *Drosophila melanogaster* heterochromatic genes

**DOI:** 10.1186/s13072-020-00358-4

**Published:** 2020-10-07

**Authors:** Parna Saha, Divya Tej Sowpati, Mamilla Soujanya, Ishanee Srivastava, Rakesh Kumar Mishra

**Affiliations:** 1 CSIR-Centre for Cellular and Molecular Biology, Hyderabad, 500007 India; 2 Academy of Scientific and Innovative Research (AcSIR), New Delhi, India

**Keywords:** Heterochromatic genes, *Drosophila melanogaster*, Pericentromeres, 5C, HP1a, Su(var)3-9, dMES-4, dADD1, H3K9me3, Het TADs

## Abstract

**Background:**

Transcription of genes residing within constitutive heterochromatin is paradoxical to the tenets of epigenetic code. The regulatory mechanisms of *Drosophila melanogaster* heterochromatic gene transcription remain largely unknown. Emerging evidence suggests that genome organization and transcriptional regulation are inter-linked. However, the pericentromeric genome organization is relatively less studied. Therefore, we sought to characterize the pericentromeric genome organization and understand how this organization along with the pericentromeric factors influences heterochromatic gene expression.

**Results:**

Here, we characterized the pericentromeric genome organization in *Drosophila melanogaster* using 5C sequencing. Heterochromatic **t**opologically **a**ssociating **d**omains (Het TADs) correlate with distinct epigenomic domains of active and repressed heterochromatic genes at the pericentromeres. These genes are known to depend on the heterochromatic landscape for their expression. However, HP1a or Su(var)3-9 RNAi has minimal effects on heterochromatic gene expression, despite causing significant changes in the global Het TAD organization. Probing further into this observation, we report the role of two other chromatin proteins enriched at the pericentromeres-dMES-4 and dADD1 in regulating the expression of a subset of heterochromatic genes.

**Conclusions:**

Distinct pericentromeric genome organization and chromatin landscapes maintained by the interplay of heterochromatic factors (HP1a, H3K9me3, dMES-4 and dADD1) are sufficient to support heterochromatic gene expression despite the loss of global Het TAD structure. These findings open new avenues for future investigations into the mechanisms of heterochromatic gene expression.

## Background

The expression of genes present in the constitutive heterochromatin is counterintuitive, and their regulatory mechanisms remain elusive. Such genes are called the heterochromatic genes due to their centromere-proximal location, presence of repressive histone marks and dependence on the heterochromatic *trans* factors for expression. Heterochromatic genes across various species include the pericentromeric genes of *Drosophila melanogaster* [1, 2]; juxtacentromeric and X-chromosome inactivation escapee genes in mammals [3–6], and centromeric genes in the knob regions of *Arabidopsis* genome [7, 8]. Of these, *Drosophila melanogaster* heterochromatic genes are one of the best-studied examples [9]. This is due to the strong genetic toolkit of the fly model system that facilitated studies of heterochromatic sequences even before the advent of high-throughput sequencing technologies. Heterochromatic genes were identified by complementation analyses using chromosomal rearrangements [10] and later by mapping these genes using fluorescence in situ hybridization to pericentromeric regions [11]. These heterochromatic genes encode proteins involved in various cellular processes, for example *light*—transporter protein; *rolled*—MAP kinase involved in imaginal disc development; *concertina*—gastrulation protein; *Nipped*-*A, Nipped*-*B*—transcription regulators, ribosomal proteins like *RpL5/15/38* and many more that remain functionally uncharacterized.

The earliest evidence that linked heterochromatic gene expression to their genomic context was reciprocal position effect variegation (PEV) [12, 13]. It was observed that the extent of variegation of these genes is correlated to the site of the breakpoint; centromere-proximal heterochromatic genes were almost unaffected while centromere-distal heterochromatic genes showed the most severe phenotypes upon translocation [14]. The abrogation of heterochromatic gene expression upon chromosomal translocation into the euchromatin [14–16], or the depletion of heterochromatic factors [17, 18], suggests that the genome organization and local concentration of certain heterochromatic *trans* factors might play a crucial role in their expression. These genes have been moved into the heterochromatin in the course of drosophilid evolution [19, 20]. However, the mechanisms by which they adapted to stay active in the repressive chromatin environment remains unclear.

The epigenetic landscape of heterochromatic genes is rather unique with the combinatorial occurrences of active and inactive histone modifications like H3K4me3/1, H3K9/27ac at the TSS and H3K9me3, H3K36me3 and HP1a on the gene body [21, 22]. These unique combinations of histone marks are hypothesized to differentially regulate heterochromatic gene transcription from the surrounding repressive pericentromeric heterochromatin (PCH). With the recent surge in the genome organization studies, the interrelatedness of structural partitioning of genome and transcriptional outputs is gaining considerable appreciation [23, 24]. In *Drosophila*, the influence of genome organization and chromatin domains on the gene expression in various developmental contexts is well studied [25]. However, there is limited understanding of how the pericentromeric genome organization, and its epigenetic landscape contribute to the transcription of heterochromatic genes.

Despite having clues about a few candidate heterochromatic genes, a well-validated model of heterochromatic gene expression in *Drosophila* is lacking. Our study focuses on filling this lacuna and gaining a global understanding of this intriguing biological phenomenon. Here, we sought to dissect the interdependence of the pericentromeric epigenetic landscape and genome organization in heterochromatic gene regulation. We present the first report of the long-range DNA interactome of PCH in flies and characterize the pericentromeric Het TADs with respect to several genomic and epigenomic features. We report that this structural organization correlates with the expression patterns of the heterochromatic genes. Depletion of heterochromatic proteins (HP1a and Su(var)3-9) disrupts the global pericentromeric genome organization but heterochromatic gene expression is minimally affected. Additionally, we investigate the roles of two other chromatin proteins-dMES-4 and dADD1, whose contribution has not been reported so far in this context. Taken together, our findings show that local DNA interactions are sufficient to maintain heterochromatic gene expression in the presence of transcriptionally favorable chromatin landscape within the pericentromeric heterochromatin.

## Results

### Pericentromeric heterochromatin is organized into distinct TADs

**Fig. 1:**
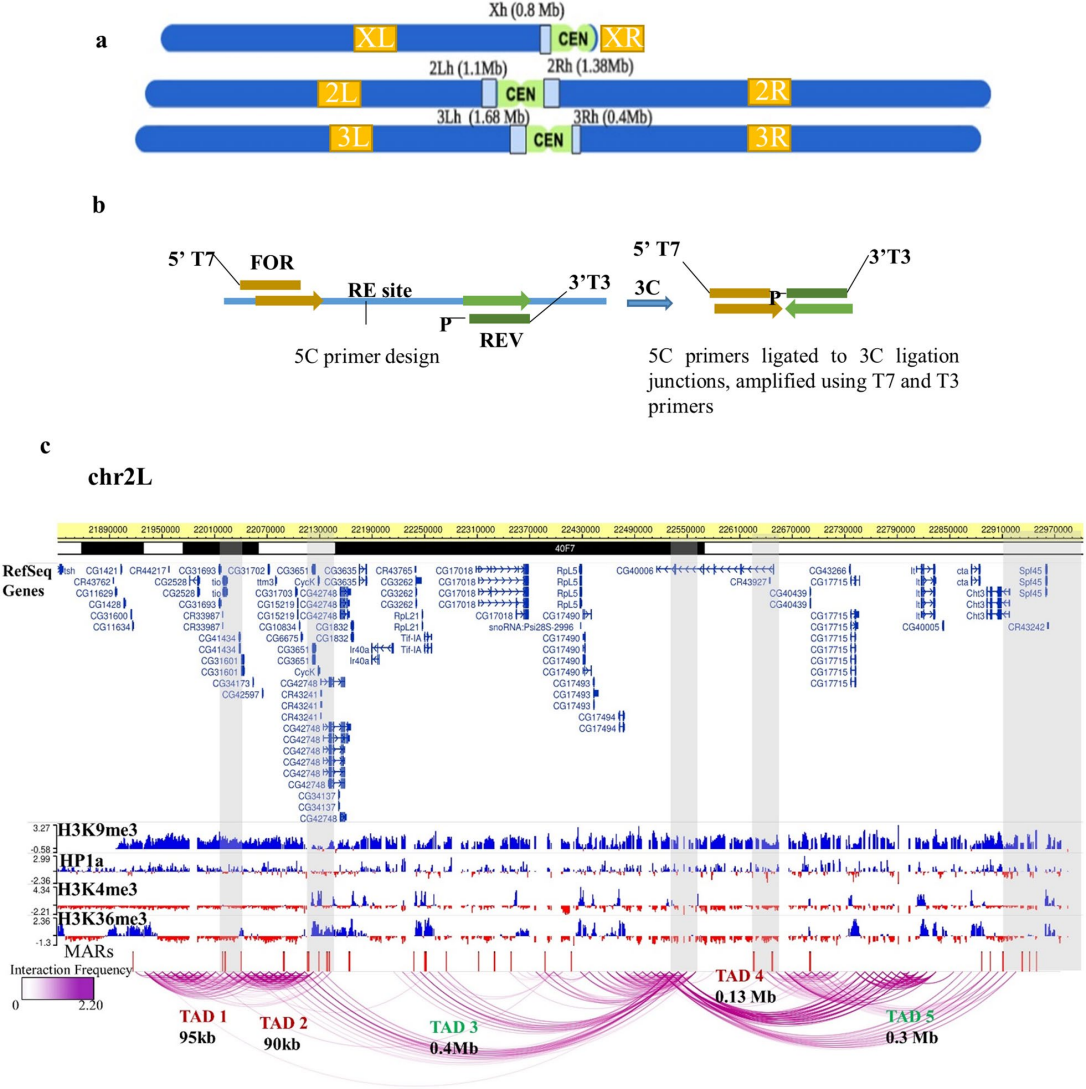
Pericentromeric heterochromatin is organized into domains **a** schematic of *Drosophila melanogaster* chromosomes showing the pericentromeric regions (light blue) included in the 5C experiment. The centromere is in green and the euchromatic regions in dark blue. **b** Schematic representation of 5C primer design by alternating primer design scheme, at the adjacent restriction enzyme sites. Forward (with T7 overhang) and reverse (with T3 overhang) primers are used to amplify the interacting DNA junctions from the 3C library. **c** A pairwise interaction map of Chr2L showing the mapped active Het TADs green and inactive Het TADs in red, respectively, along with the epigenetic marks of H3K9me3, HP1a, H3K4me3 and H3K36me3. Active genes in the pericentromeres are enriched for H3K9me3 along with active histone modifications. MARs are indicated by red bars. TAD boundaries (grey bars) partition distinct epigenomic domains

To investigate the pericentromeric genome organization and its contribution to heterochromatic gene expression, we mapped the long-range DNA interactome in the *Drosophila melanogaster* PCH. The exhaustive information of chromatin contacts in the heterochromatin is often limited in existing genome-wide analyses (Additional file 1: Fig. S1). Therefore, we performed chromosome conformation capture carbon copy (5C) targeted at the pericentromeric regions (as per the annotation of euchromatic–heterochromatic junctions in the S2 cells [26, 27]) as shown in Fig. 1a. 678 primers (Additional File 2) were designed at the adjacent EcoRI sites, Fig. 1b, to interrogate the pericentromeric regions on chr2L, chr2R, chr3L, chr3R and chrX that covered 6 Mb of the *Drosophila melanogaster* genome and approximately 170 genes. chr4 was excluded as the heterochromatin demarcation at this genomic locus is unclear. The 3C and 5C libraries were prepared from S2 cells with appropriate quality controls (Additional file 1: Fig. S2a-b). We selected paired-end reads with the EcoRI site, which were further filtered to remove the low-quality reads (Additional file 3).

Next, we proceeded to computationally define the TADs using Directionality Index scores across the biological replicates (Additional file 1: Fig. S3). TAD boundaries were obtained using the Directionality Index method described previously [28, 29]. The correlation between the replicates was calculated and consensus TADs were derived using a Hidden Markov Model (HMM) [29] (See “Materials and methods”). We report 21 Het TADs (5 on chr2L, 6 on chr2R, 6 on chr3L, 4 on chrX) with TAD sizes of 90 kb–0.3 Mb and TAD border sizes in the range of 10–40 kb (Additional file 1: Table S1). The number of interactions mapped to chr3R was not enough to demarcate TADs computationally. Some of the interactions reported in the 5C-seq (6 out of 10) were further validated using 3C PCR (Additional file 1: Fig. S4).

A closer inspection of the intrachromosomal interactions reveals several local domains of interacting regions along the pericentromere, that correlate with specific epigenetic signatures—active (enriched for both H3K9me3, HP1a and H3K4me3, H3K36me3 marks) and repressive/inactive (H3K9me3, HP1a marks only) Het TADs, as shown in Fig. 1c and Additional file 1: Fig. S5a–d. The TAD borders mark the transition from one Het-TAD to another, in terms of compartmentalization of the long-range interactions and in certain cases (7 out of 20 TAD borders), also demarcating functionally distinct epigenomic (active vs inactive) domains. We also mapped several inter-chromosomal interactions amongst the PCH regions on each chromosome arm (Additional file 1: Fig. S6a). These are likely to be functionally relevant as the centromeres coalesce into chromocenters in *Drosophila* nuclei. The inter-chromosomal interactions connecting the active–active and inactive–inactive Het TADs are the most and least predominant, respectively (Additional file 1: Fig. S6a). The repertoire of long-range DNA interactions obtained provides the first report of the presence of distinct higher order genomic architecture within the condensed *Drosophila melanogaster* chromocenter.

### Characteristics of the heterochromatic TADs

To characterize the Het TADs, we compared the distribution of various genomic and epigenomic features encompassing the intra-TAD regions (within the TADs) and TAD borders. Intra-TAD regions were scaled to 150 kb (TAD size ranging from 80 kb to 0.4 Mb), binned at 30 kb and TAD borders were scaled to 30 kb (TAD borders ranging from 10 kb to 40 kb), binned at 10 kb, respectively. The features included were (a) architectural/insulator-binding proteins (IBPs)—dCTCF, BEAF32, GAF, CP190, Su(Hw), mod(mdg4), (b) genomic features—(1) nuclear matrix-associated regions (MARs), mapped to the pericentromeric regions in *Drosophila* S2 cells (Additional File 4), (2) predicted boundary elements (BEs) from the cdBEST tool [30] and enhancers (STARR-seq) [31]; and c) epigenomic features like histone modifications and heterochromatin proteins (HP1). The overlapPerm test was used to statistically compare (using *p* value and Z-score) the overlap of these features with TAD borders and randomized genomic regions, to rule out associations that might occur by random chance.

**Fig. 2:**
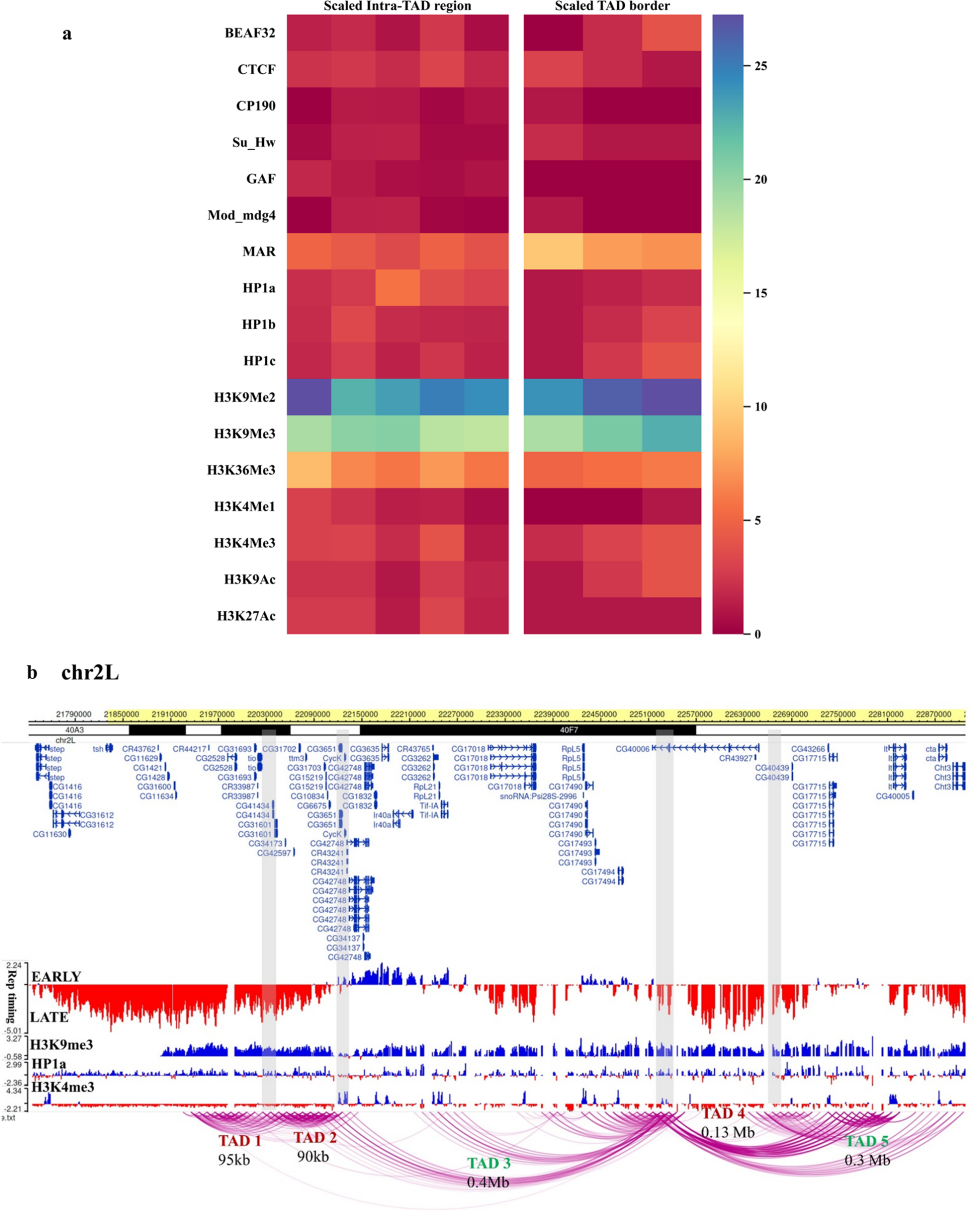
Characteristics of the pericentromeric Het TADs **a** comparative heatmap for the enrichment of various genomic and epigenomic features across scaled average Het TAD borders (30 kb) and intra-TAD regions (150 kb). TAD borders are enriched in MARs and BEAF32 while other architectural proteins are present predominantly at the intra-TAD interactions (for the *p* values of the overlap of features with TAD borders, see Additional file 1: Figure S5 and S6). Active histone modifications, H3K4me1, H3K4me3, H3K9ac are present within the TADs, whereas H3K36me3 and heterochromatic marks—H3K9me2/3 and HP1a—are present both at the intra-TAD and TAD border regions. **b** Representative snapshot showing the overlap of Het TAD organization in chr 2L with the published replication timing domains in S2 cells—late-replicating regions coincide with inactive Het TADs while active Het TADs have both early- and late-replicating regions within them

We analyzed the distribution of various architectural and insulator protein binding sites that are known to mediate long-range DNA interactions in flies [32]. Figure 2a shows a heatmap comparing the relative distribution of the occupancy of architectural proteins and histone marks across scaled intra-TAD and TAD border regions. The TAD borders are enriched in BEAF32 while GAF and mod(mdg4) are present in the intra-TAD regions (Additional file 1: Fig. S7). dCTCF, a well-known architectural protein, CP190 and Su(Hw) have comparable occupancy at both the intra-TAD and TAD border regions.

MARs are regions of the genome that tether a genomic locus to the ribonucleoproteinaceous substratum of the nuclear matrix. They are proposed to act as scaffolds for various nuclear processes [33], and assist in the organization of the genome (via DNA-looping), thereby modulating gene expression [34, 35]. The MAR data of S2 cells used in this study were obtained as per the protocol in [36]. The overlap of Het TAD borders with MARs was highly significant (*p* value 0.001) in comparison to the intra-TAD regions (Fig. 2a and Additional file 1: Fig. S8a). On the contrary, enhancers are less abundant at the TAD borders (*p* value 0.34, Additional file 1: Fig. S8b), indicating that they are more likely to influence intra-TAD interactions.

H3K4me1 and H3K27ac are enriched in the intra-TAD regions while H3K4me3 and H3K9ac are comparable for both the regions. Interestingly, H3K36me3 is also enriched within the TADs where most heterochromatic genes reside. H3K9me2/3 are present both at the TAD borders and intra-TAD regions. Inter-chromosomal interactions mapped in our study are also enriched for MARs followed by various combinations of insulator-binding proteins, predominantly BEAF32 and dCTCF (Additional file 1: Fig. S6b).

Given that heterochromatic regions are enriched in various transposable elements and repeats, we investigated their role in pericentromeric genome organization. We chose all available classes of repeat elements from UCSC genome browser and overlapped them with the TAD borders identified in our study. The comparative heatmap (Additional file 1: Fig. S9) shows no difference in the distribution of repeat features between the intra-TAD regions and the TAD borders. Thus, there is no apparent functional correlation of the repeat occurrence with pericentromeric genome organization. We also overlapped the 5C interaction domains with replication timing data from S2 cells [37]. As shown in Fig. 2b, we find active Het TADs encompass both the early- and late-replicating regions, where early replicating regions generally coincide with active heterochromatic genes. Inactive Het TADs are expectedly rich in late-replicating regions, except for Chr X (Additional file 1: Fig S10a-c). This could be a sex-specific characteristic since the S2 cells are derived from male embryos.

**Fig. 3:**
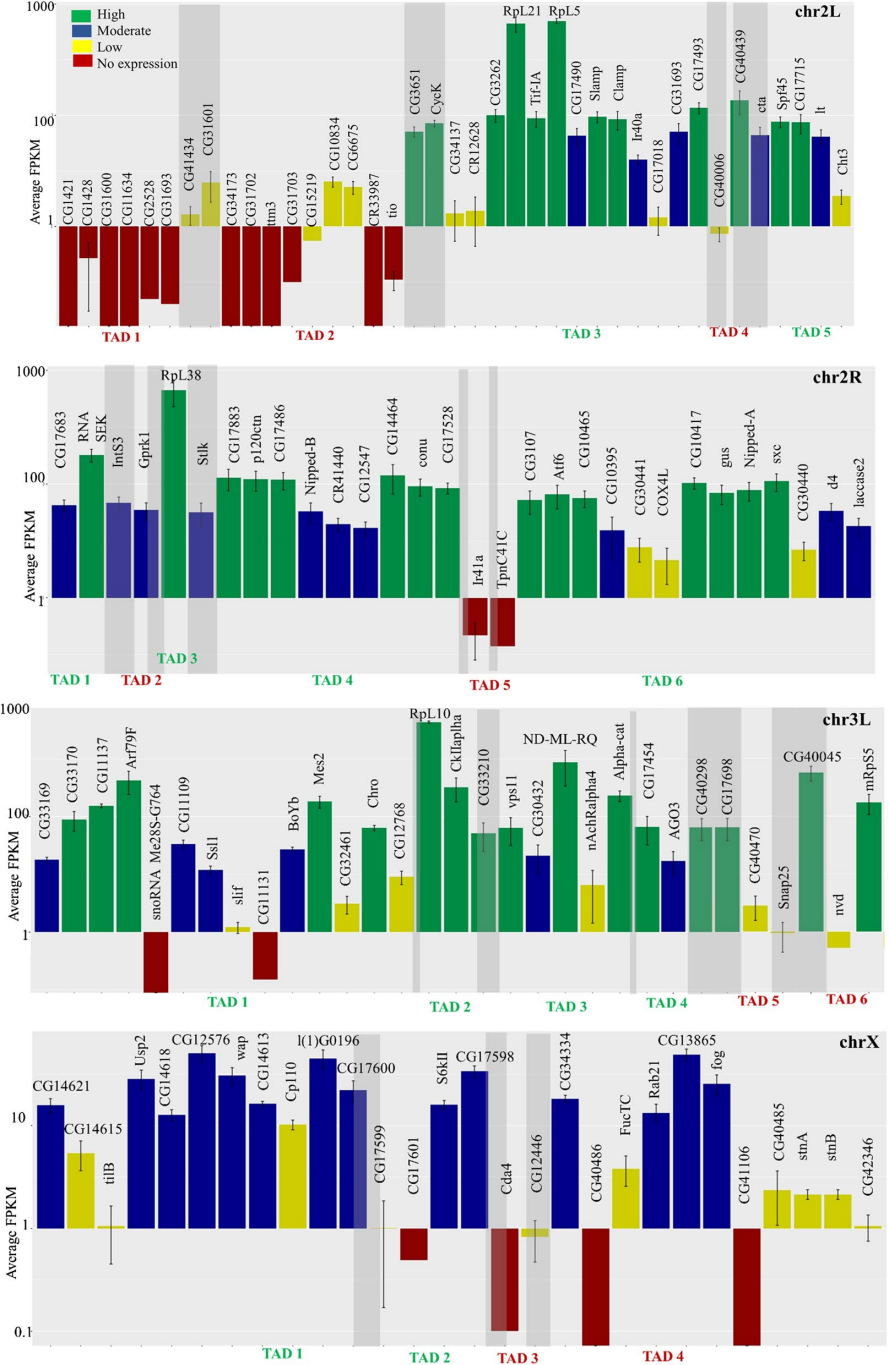
Correlation between pericentromeric genome organization and heterochromatic gene expression—bar plots showing the expression data (average FPKM values + SE, *n* = 3) for each of the heterochromatic genes on the Het regions of chromosome arms compared with the TAD structure of chr 2L, 2R, 3L and X. Genes are colour-coded according to the expression levels. TAD borders are indicated in grey bars, some of which fall in the intergenic regions or have genes spanning the entire border region. The expanse of the TADs does not correlate to the actual mapped TAD sizes and only shows the number of genes in a particular Het TAD

TADs reported in *Drosophila melanogaster* have been shown to overlap with different epigenetic and thus expression domains [38]. Therefore, we asked whether the pericentromeric genome organization into Het TADs is related to heterochromatic gene expression. To this end, we performed a total RNA sequencing of S2 cells and overlapped the transcriptomic data with the Het TADs identified in our study. We find that most Het TADs encompass genes with similar expression levels (Fig. 3). The gene activity status of most Het TADs is also substantiated by the presence/absence of RNA Pol II peaks within them (Additional file 1: Fig S11). Exceptions to the trend of similarly expressing genes in a Het TAD include chr 2L TAD4, chr 2R-TAD2 which have genes extending into adjacent TADs and, chr 3L TAD 1, chrX TAD 2 and 4 where the expression levels are highly variable (Fig. 3, Additional file 1: Fig. S12). Although we do not have the TAD information for chr3R, we do find both active and silent heterochromatic genes on this chromosome arm (Additional file 1: Fig S5d and S12). Taken together, the higher order genome organization into Het TADs, epigenetic signatures within them and the expression status of the heterochromatic genes are well correlated.

### Loss of heterochromatic factors weakens Het TAD insulation but does not affect heterochromatic gene expression

Genetic studies using a candidate-based approach have shown that heterochromatic genes depend on the repressive chromatin environment for their expression [17, 18]. In flies, HP1a and Su(var)3-9 (the major H3K9 methyltransferase) are the two major players involved in the maintenance of heterochromatin structure and function [39]. Therefore, we investigated the effects of depletion of HP1a or Su(var)3-9 on pericentromeric genome organization and heterochromatic gene expression. Additionally, it would also reveal whether the pericentromeric TAD structure is functionally correlated with the expression of genes within them.

**Fig. 4:**
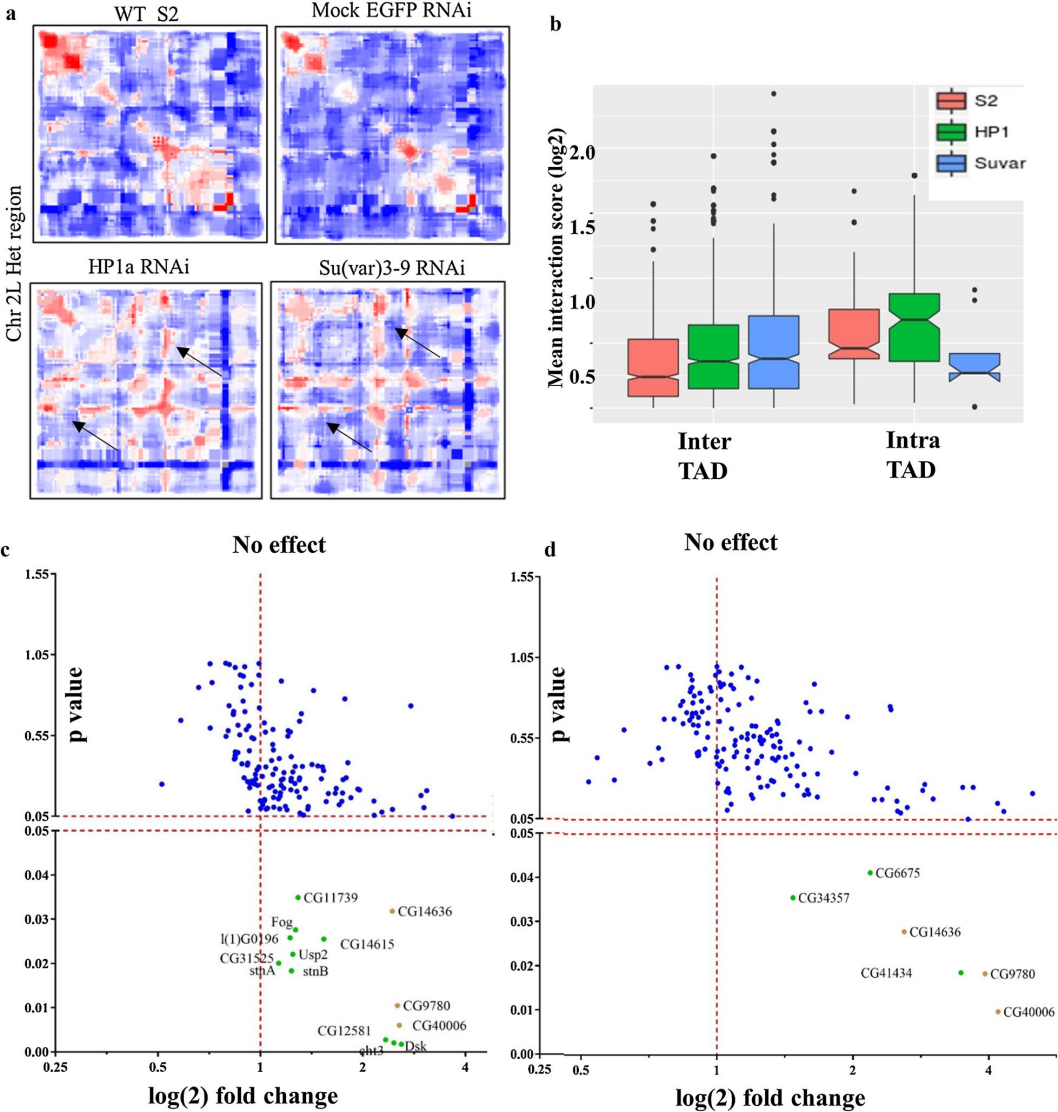
HP1a and Su(var)3-9 RNAi results in loss of TAD insulation with minimal effect on heterochromatic gene expression a) comparison of the heatmaps across the heterochromatic regions of chr 2L included in the 5C seq in WT, mock RNAi, HP1a RNAi and Su(var)3-9 RNAi-treated cells. **a** The increase in inter-TAD interactions is evident from the increase in longer range interactions (black arrows) with high interaction frequency on either side of the diagonal of the heatmap. **b** Quantification of the effect of knockdown of HP1a and Su(var)3-9 on the inter and intra-TAD interaction frequencies using notched box plot where the notch indicates the median value at 95% confidence interval. The median notch of the WT and HP1a RNAi or Su(var)3-9 RNAi conditions do not coincide indicating the gain of new interactions, with high interaction scores (including the outliers). **c** Scatter plot showing the effect of HP1a and d) Su(var)3-9 knockdown on the expression levels of heterochromatic genes (*n* = 3). Differentially expressed (Student’s *T* test; *p* value < 0.05) genes are labeled in green. DEGs common between HP1a and Su(var)3-9 RNAi are marked in yellow

We performed RNAi-mediated knockdown of HP1a in S2 cells that decreased endogenous HP1a protein levels by ~ 72%. Parallelly, we also performed Su(var)3-9 RNAi in another population of S2 cells resulting in a ~ 70% decrease of endogenous Su(var)3-9 levels (Additional file 1: Fig. S13a). Following this, we performed 5C-seq on HP1a RNAi and Su(var)3-9 RNAi-treated cells. Generally, inter-TAD interactions along chromosome arms are less frequent and have low interaction scores. Compared to the WT and mock RNAi controls, the HP1a or Su(var)3-9 depleted cells showed an increase in inter-TAD interactions (interactions further away from the diagonal on the heatmap) with higher interaction scores, as seen in Fig. 4a and Additional file 1: Fig. S13b. Stronger inter-TAD interactions indicate that depletion of HP1a and Su(var)3-9 weakens TAD insulation.

There is also a significant increase in intra-TAD interactions in HP1a RNAi condition (Fig. 4b). On the other hand, the intra-TAD interactions show a lower average interaction score in Su(var)3-9 RNAi as compared to the mock RNAi, indicating that HP1a and Su(var)3-9 have distinct roles in regulating intra-TAD organization. Overall, the knockdown of HP1a or Su(var)3-9 predominantly affects global inter-TAD organization in the pericentromeres, while the local (intra-TAD) interactions are affected to different extents in the two RNAi conditions.

Next, we asked whether the disruption of TAD structure upon HP1a RNAi or Su(var)3-9 RNAi affects the expression of heterochromatic genes. To this end, we performed transcriptomic analyses on the HP1a RNAi and Su(var)3-9 RNAi-treated cells. Globally, 281 and 216 genes are differentially expressed in HP1a RNAi and Su(var)3-9 RNAi conditions respectively, and 101 differentially regulated genes are common between the two conditions (Fig S14a-b, Additional file 5). This is in line with the previous reports where both euchromatic and heterochromatic genes were shown to be affected by the depletion of heterochromatic proteins [40].

Counterintuitively, heterochromatic gene expression is only subtly affected in both the knockdown conditions. A small subset of 20 and 9 heterochromatic genes was differentially expressed in HP1a RNAi and Su(var)RNAi, respectively, which also include certain non-coding transcripts like *CR41501, CR33294, 18SrRNA*-*Psi: CR41602*, as shown in Fig. 4c–d and Additional file 1: Table S2a. The number of heterochromatic genes affected by the Su(var)3-9 KD were less than those in the case of HP1a KD. This prompted us to investigate the levels of other H3K9 methyltransferases like G9a and Setdb1 (Additional file 1: Table S2b). G9a has levels comparable to WT in both the knockdown conditions. Setdb1 is upregulated and could have compensated for the depletion of Su(var)3-9 expression. This along with the functional non-redundancy of HP1a with HP1b and/or HP1c might explain why more heterochromatic genes are affected in HP1a vs Su(var)3-9 KD conditions. However, in both the cases of HP1a RNAi- and Su(var)3-9 RNAi-mediated perturbed PCH TAD organization, global TAD organization seems dispensable for the maintenance of heterochromatic gene expression.

### dMES-4 and dADD1 are novel chromatin factors in the regulation of heterochromatic gene expression

**Fig. 5:**
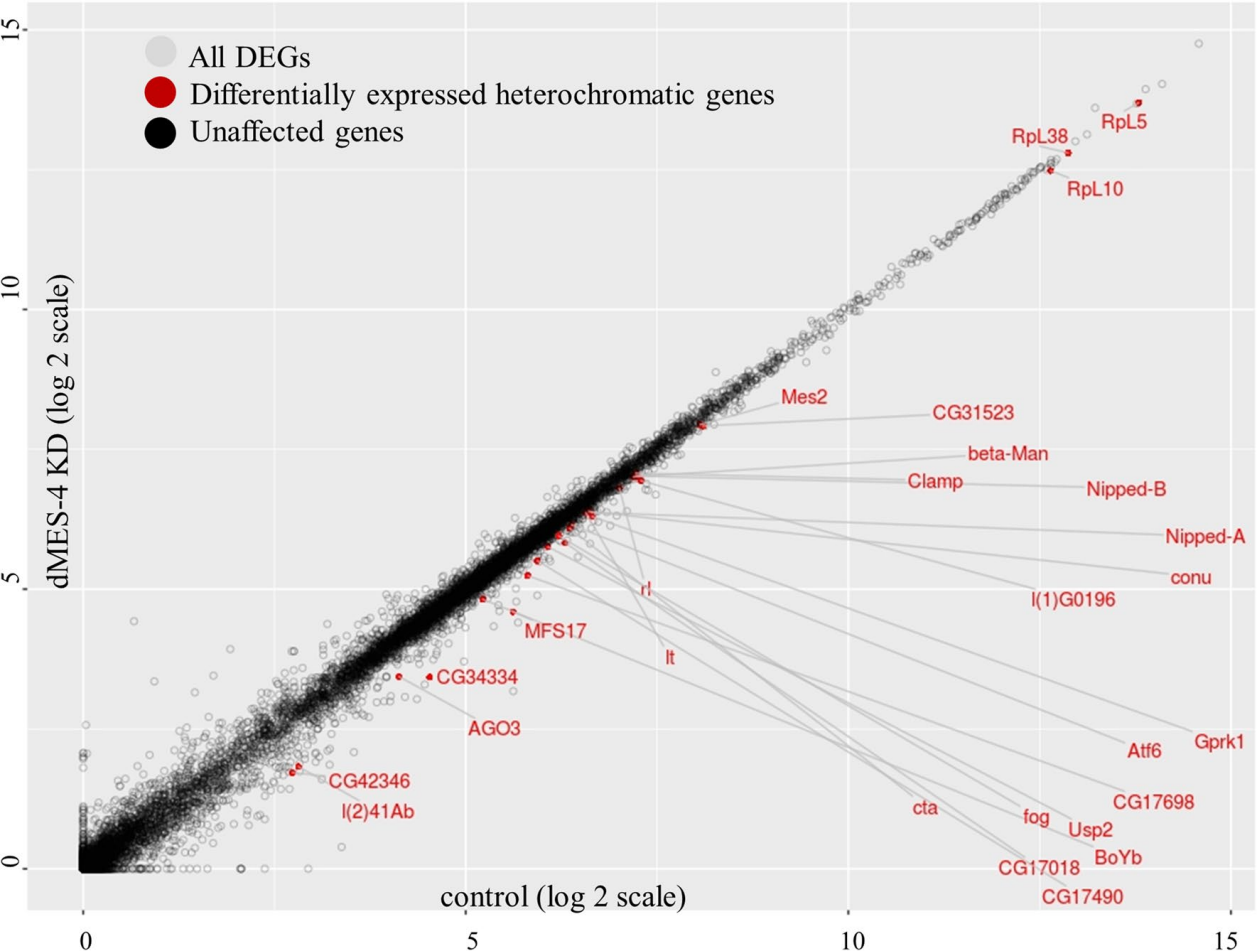
dMES-4 knockdown downregulates the expression of a subset of heterochromatic genes—scatter plot showing the effect of dMES-4 knockdown on S2 cell transcriptome—comparison of WT vs dMES-4 KD. Control RNA seq data on X-axis and dMES-4 knockdown RNA seq data on *Y*-axis. Differentially expressed (*p* value < 0.05) heterochromatic genes are in red

Our results show that HP1a or Su(var)3-9 KD has minimal effect on heterochromatic gene expression. Therefore, we sought to identify other pericentromeric factors that regulate heterochromatic gene expression. Earlier reports have suggested that heterochromatic genes are marked by both H3K9me3 and H3K36me3 [21], and H3K36me3 is indeed present in the active Het TADs (Fig. 1c). This is an interesting combination of functionally opposite histone modifications; whose functional relevance is unknown. Therefore, we analyzed the role of dMES-4, a H3K36 methyltransferase predominant in PCH [41], in heterochromatic gene expression. In this regard, we analyzed the publicly available transcriptomic data for knockdown of dMES-4. We reanalyzed the data only for the heterochromatic genes both on the chromosome arms and unassembled heterochromatin. We find that depletion of dMES-4 results in significant downregulation of 27 heterochromatic genes (FDR-adjusted *p* value < 0.05), as shown in Fig. 5. However, many of these significantly differentially expressed heterochromatic genes show only a slight downregulation, with < 1.5-fold change in expression (Additional file 6). These small yet significant changes in expression may have phenotypic consequences in the organism. Genes differentially expressed upon dMES-4 KD include the well-studied heterochromatic genes like *light, rolled, Nipped*-*A* and more importantly, none of the heterochromatic genes were found to be upregulated.

**Fig. 6:**
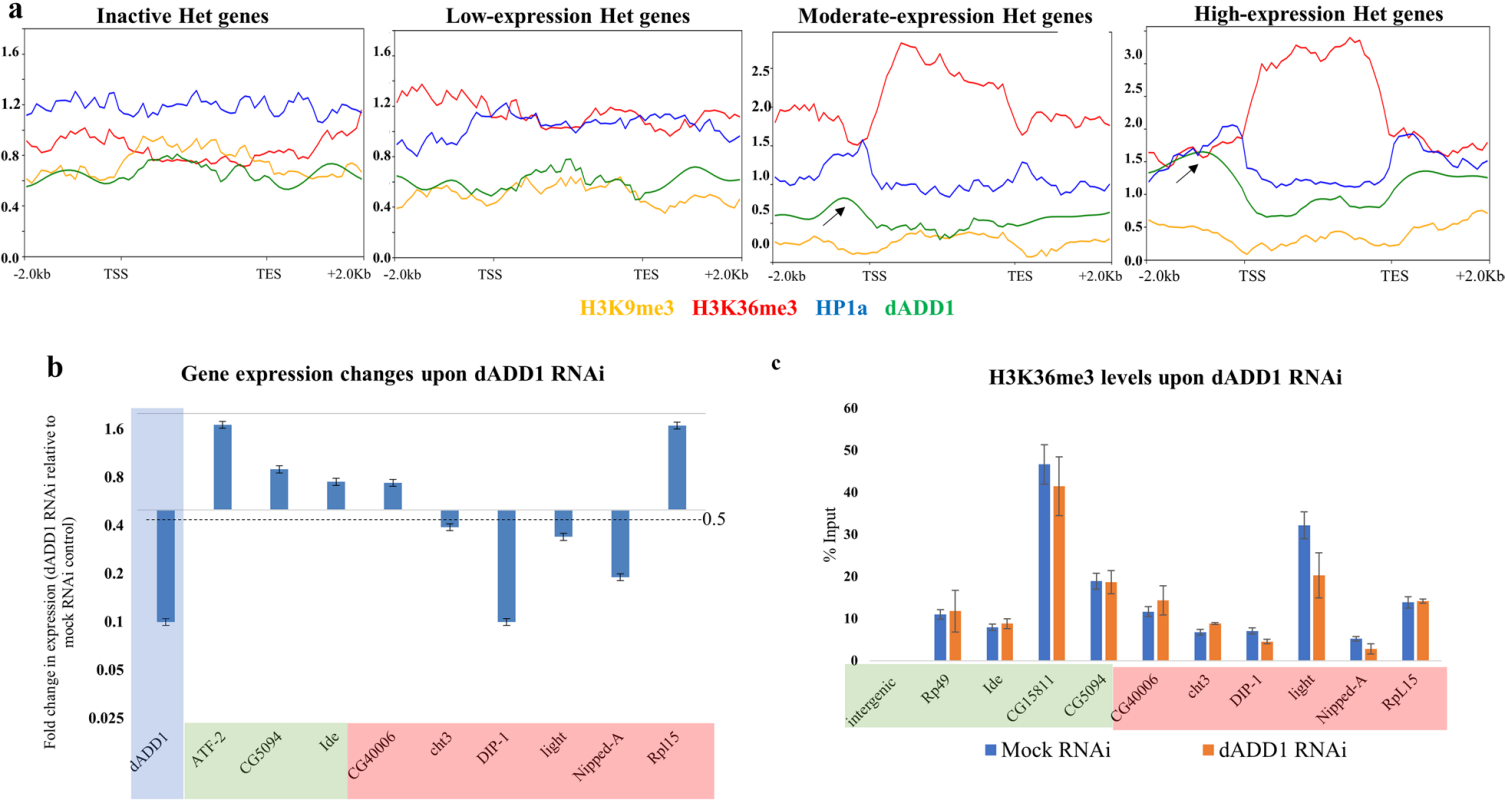
Distribution of dADD1 on heterochromatic genes and effects of RNAi-mediated dADD1 knockdown **a** Comparison between the expression levels of heterochromatic genes and occupancy of H3K9me3, H3K36me3, HP1a and ADD1 on the gene body across heterochromatic genes with no, low, moderate and high expression. Heterochromatic gene expression is associated with an increased enrichment of dADD1 at the TSS and H3K36me3 at the gene body. **b** RT-qPCR data showing the fold change in expression (normalized to *Rp49*) upon RNAi-mediated dADD1 knockdown compared to the control (*n* = 2, Mean + SE). Euchromatic genes in green and heterochromatic genes in red. **c** Comparison of the enrichment of H3K36me3 marks uisng ChIP-qPCR in WT and dADD1 knockdown conditions (*n* = 2, Mean + SE)

We also chose to study the chromatin remodeler dADD1 (CG8290) as a potential regulatory factor. dADD1 has been shown to interact with both H3K9me3 and HP1a, co-localizes at the pericentromeres [21], and, therefore, might regulate heterochromatic gene expression in the presence of either or both HP1a and H3K9me3 marks. To understand how does dADD1 occupancy on the heterochromatic genes correlates with their expression, we grouped all the heterochromatic genes into four categories a) no-expression (< 1 FPKM) b) low expression (1-10 FPKM) c) moderate expression (11-50 FPKM) d) high expression (> 51 FPKM). We used the available ChIP-seq data of dADD1 to compare its enrichment on each set of these genes along with the occupancy data of H3K9me3, H3K36me3 and HP1a. Interestingly, we find that for “No” and “Low expression” heterochromatic genes, there is minimal enrichment of the dADD1 and H3K36me3 marks at the promoters and the gene body, respectively. On the contrary, there is increased binding of dADD1 upstream of the TSS, concomitant with an increased H3K36me3 binding at the gene bodies of the “moderate” and “high” expression heterochromatic genes (Fig. 6a). This trend indicated that dADD1 could have a functional role in regulating heterochromatic gene expression, as has also been previously suggested [41].

To confirm whether dADD1 binding at the promoter of expressed heterochromatic genes has functional consequence on their expression, we performed RNAi-mediated knockdown of dADD1 in S2 cells using dsRNA that targets all the three dADD1 isoforms. We confirmed the knockdown of ADD1 levels using RT-qPCR (Fig. 6b). While euchromatic genes are unaffected (*CG5094* and *Ide*) or upregulated (*ATF*-*2*), the levels of a subset of heterochromatic genes (*DIP1, light, Nipped*-*A*) are lowered (Fig. 6b). *CG40006* is affected upon both HP1a and Su(var)3-9 knockdown and *Cht3* in HP1a knockdown, but neither is affected in case of dADD1 knockdown. Based on the trend in Fig. 6a, we investigated the levels of H3K36me3 marks upon dADD1, using ChIP-qPCR for H3K36me3 marks (Fig. 6c). The levels of H3K36me3 on the heterochromatic genes affected by dADD1 knockdown are lowered in case of *light* and to a lesser extent in the case of *DIP1 and Nipped*-*A.* These genes also get downregulated upon dMES-4 knockdown (Fig. 5). Transcript levels of heterochromatic gene *RpL15* are upregulated but deposition of H3K36me3 marks is unaffected by dADD1 KD. This indicates that not all heterochromatic genes are regulated by the same factors. dADD1 has been widely studied in the context of heterochromatin maintenance [42, 43]. Our findings lead us to conclude that dADD1 is also an important player in regulating heterochromatic gene expression, thereby supporting the hypothesis that pericentromeric factors are indeed essential for the expression of a subset of heterochromatic genes.

## Discussion

### *Drosophila melanogaster* pericentromeres are organized into discrete TADs

One of the key findings of our study is the map of long-range DNA interactome in the PCH. We find that there exists a discrete TAD organization compartmentalizing the local chromatin interactions within the previously reported “HP1a/centromeric” mega-domains [38]. In line with the reports for the global TAD organization in *Drosophila* [32], we find that BEAF32 is enriched at the Het TAD borders but not dCTCF. Intra-TAD interactions are marked by GAF. In flies, GAF has also been shown to mediate gene activation [44]. Given the prevailing skepticism in the field with regard to nuclear matrix-associated regions, further experimental investigations are required to validate the functional significance of the association of Het TAD borders with MARs.

Intra-TAD regions are also enriched for enhancer marks like H3K27ac and H3K4me1 and active transcriptional elongation mark of H3K36me3 supporting the presence of actively expressing heterochromatic genes from active Het TADs. Although there are speculations of repeat elements playing a role in the genome architecture of pericentromeric regions, they show similar enrichment at both the Het TAD borders and intra-TAD regions. Many of the intra-TAD and inter-chromosomal interactions were found to map to the enhancers obtained from the STARR-seq, indicating that they are most likely functional enhancer-promoter contacts [31]. The functional correlation of these features and PCH TAD organization would require further studies aimed at deleting candidate regions in the genome and looking at the changes in genome organization and consequently, heterochromatic gene expression.

Interestingly, we find that most heterochromatic genes within a Het TAD have similar expression levels that also correspond to the epigenetic signatures of that TAD. This indicates that these genes are likely to share the same core regulatory network of long-range interactions. However, the heterochromatic genes within a TAD do not share any common gene ontology. A subset of genes located at the TAD borders (19 genes) show high to medium levels of expression, like the trend reported for the euchromatic TAD borders [32]. Pericentromere-associated domains have so far been reported only in mice [45]. Given the dearth of information regarding the hierarchical genome organization within the pericentromeres, our findings provide a detailed characterization of the *Drosophila melanogaster* PCH domains, which set the stage for further probing into its functional role in heterochromatic gene expression.

### Pericentromeric genome organization changes caused by HP1a/Su(var)3-9 RNAi has minimal effect on heterochromatic gene expression

Classical genetics experiments have provided us with insights into the role of two heterochromatic factors, HP1a (Su(var)205) and the H3K9me3 methyltransferase Su(var)3-9, in the regulation of heterochromatic gene expression [15, 18]. However, a detailed understanding of their genome-wide effects on these genes has not been reported. Upon knocking down these two proteins in S2 cells, we find that the pericentromeric genome organization is perturbed, as marked by increased inter-TAD interactions. At the transcriptomic level, we find that HP1a RNAi and Su(var)3-9 RNAi affect several genes genome wide. Notable examples of upregulated genes are *Trl (GAF)*, *Rad50, HmgZ, Nup75, Su(var)2*-*10*, snRNA/snoRNA genes and downregulated genes are *LamC* and heat shock proteins respectively, some of which are known interactors or targets of the two proteins. However, HP1a RNAi and Su(var)3-9 RNAi did not affect the expression of levels of the majority of the heterochromatic genes.

This prompted us to compare our findings with the previously reported observations of HP1a and Su(var)3-9 depletion downregulating candidate heterochromatic gene expression. Genetic studies using mutant alleles of *Su(var)205* (HP1a) had reported variegated silencing of heterochromatic genes *light* and *rolled* in mutant larvae [18]. However, the difference in RNA levels upon knockdown of HP1a leading to discernable phenotypes was only 1.5-fold. Following this, reports by Greil and colleagues showed no significant change in expression of *light* and *rolled* upon RNAi-mediated depletion of HP1a or Su(var)3-9 (90% knockdown) in Kc167 cells [46]. Our data are consistent with the findings of the second report. However, to reconcile the differences in these observations, we put forward the following rationale. First, subtle changes in a candidate heterochromatic gene expression in the genetic studies produced phenotypes at the organismal levels. Such subtle (> 1.5 fold) changes in heterochromatic gene expression captured in the genome-wide transcriptomic analyses cannot be translated to any discernable phenotypes, if any, in cell-culture systems. However, cell-culture systems are useful to capture the global genome organization and transcriptomic changes of heterochromatic genes that were not addressed in previous candidate-based approaches. Second, RNAi is transient and hence cannot achieve the 100% depletion of knockouts. Thus, the possibility that epigenetic landscape can be sustained for few cell divisions in the RNAi-treated cells to facilitate heterochromatic gene expression despite the perturbations in genome organization, cannot be ruled out. Both HP1a and Su(var)3-9 are essential genes, thus, generating complete knock-out of these two proteins makes the cells/organisms non-viable. Future endeavors to generate conditional knockouts using CRISPR based systems or conditional depletion using auxin inducible degrons shall be helpful to overcome this limitation. Third, there are reports suggesting that TAD structure in certain contexts could be dispensable for gene expression and the robustness of expression is controlled at multiple levels of genome organization [47, 48]. Along similar lines, we hypothesize that the local DNA interactome supports heterochromatic gene expression despite the remodeling of the global Het TAD structure. Lastly, in addition to the core regulatory network involving the major heterochromatin proteins like HP1a and Su(var)3-9, there are other yet to be identified factors that act in heterochromatic gene-specific contexts.

### dADD1 and dMES-4, in concert with heterochromatic factors, regulate the permissiveness of heterochromatic gene expression from the PCH

We find that dMES-4 knockdown downregulates the expression of several heterochromatic genes most of which belong to Group C/D or Group-I as per [21, 22]. Heterochromatic genes in these groups have the presence of both inactive H3K9me3, HP1a, and active H3K36me3 marks at their exons. These genes do not get affected upon single knockdown of either HP1a or Su(var)3-9 but are downregulated upon depletion of dMES-4. This indicates that the combinatorial histone modification of H3K9me3/HP1a and H3K36me3 on these genes is of functional relevance. However, it remains to be understood if the H3K36me3 mark is the cause or consequence of heterochromatic gene transcription.

Delving further into the importance of epigenomic landscape in heterochromatic gene regulation, we studied the role of dADD1-*Drosophila* homolog of human ATRX. hATRX is an ATP-dependent chromatin protein with N-terminal ADD (ATRX-DNMT3-DNMT3L) domain and belongs to the SNF2 family of chromatin remodelers. hATRX, associates with the heterochromatin by localizing with H3K9me3/H3K4me0 and its recruitment to the genes is stabilized by the formation of a tripartite interaction with HP1a and H3K9me3 [49]. In *Drosophila*, the h*ATRX* gene is split into *dADD1* (has ADD DNA binding domain but lacks ATP helicase domain) and *dXNP/ATRX* (the ATP helicase). All three dADD1 isoforms localize to the pericentromeric and telomeric heterochromatin and interact with dXNP/ATRX [42]. In vitro assays have shown that dADD1 binds to H3K9me3/H3K4me0 and interacts with HP1a and acts as a mild suppressor of variegation in flies [41]. Our results show that there is an increase in the binding of dADD1 at the TSS of moderate to highly expressing heterochromatic genes. dADD1 RNAi lowers the expression of a subset of heterochromatic genes tested. We hypothesize that dADD1 in Drosophila is possibly also responsible for remodeling the epigenome of certain active heterochromatic genes, thus making them accessible to the transcriptional machinery. dADD1 has been reported as a negative regulator of transcription; therefore, for certain heterochromatic genes, how dADD1 binding at the promoter is correlated with enhanced gene expression or H3K36me3 deposition warrants further investigations.

**Fig. 7:**
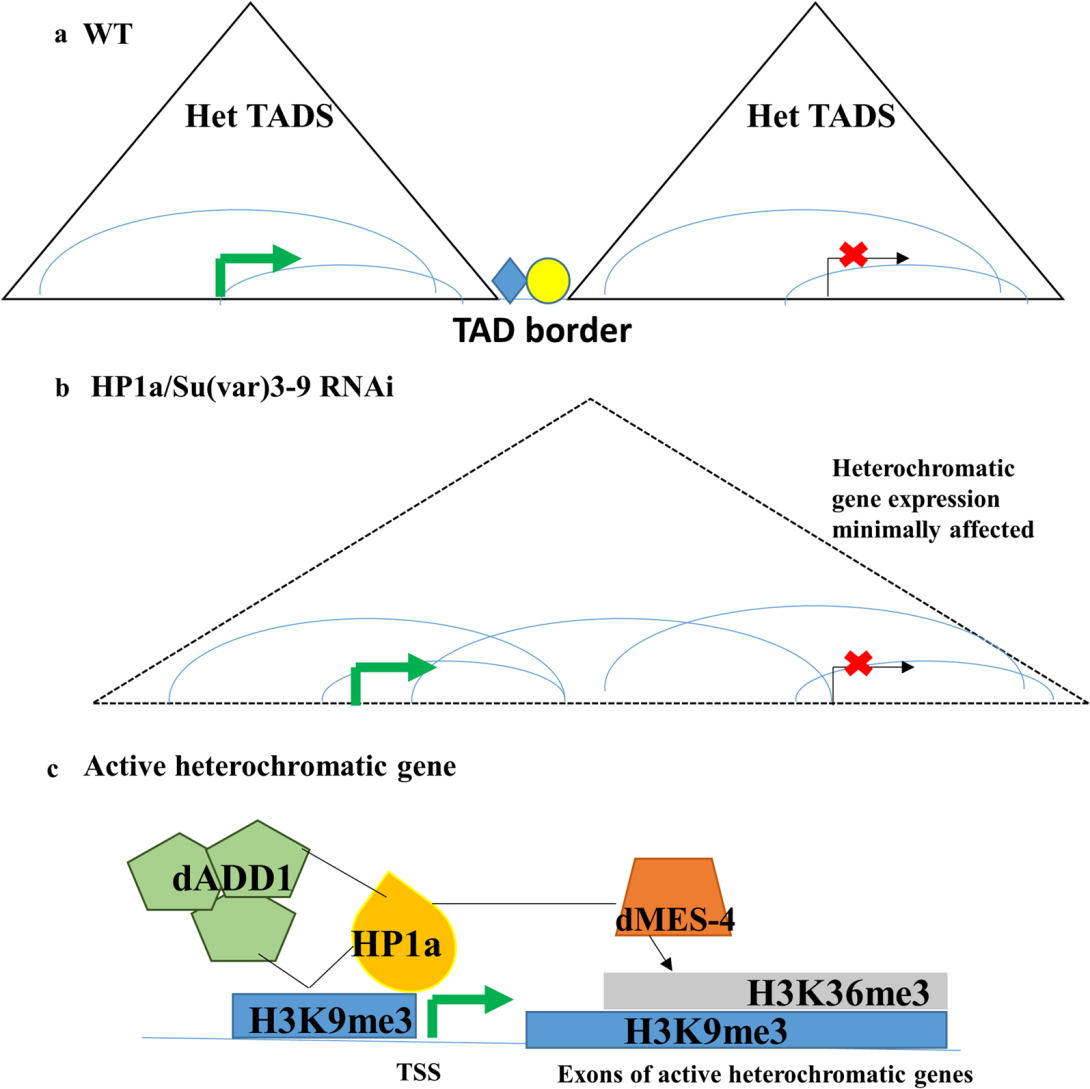
Interplay of pericentromeric genome organization and epigenetic landscape regulates heterochromatic gene expression. **a** Distinct genome organization within the pericentromeres partitions the centromeric mega-domains into active and repressed Het TADs which correlate to the expression state of the encompassed heterochromatic genes. TAD borders are enriched for architectural proteins shown in yellow and blue shapes. **b** RNAi-mediated knockdown of heterochromatic proteins like HP1a or Su(var)3-9, the global TAD structure is largely perturbed but the local intra-TAD interactions are maintained. RNAi of HP1a or Su(var)3-9 minimally affects heterochromatic gene expression. **c** *Drosophila melanogaster* active heterochromatic genes, as the name suggests, show enrichment of repressive (H3K9me3/HP1a) and active epigenetic marks like H3K36me3. dADD1, an interactor of HP1a and H3K9me3, binds to upstream of the TSS and is probably involved in the regulation of heterochromatic gene expression in concert with heterochromatic factors (H3K9me3 marks and/or HP1a). dMES-4 (H3K36 methyltransferase) enriched at the pericentromeres is plausibly responsible for the deposition of H3K36me3 marks on the gene bodies of active heterochromatic genes. Mechanistic details of regulation of heterochromatic gene expression by these factors—HP1a, Su(var)3-9, dADD1 and dMES-4 is not well understood. The combinatorial histone mark of H3K9me3/HP1a and H3K36me3 at the exons is likely to regulate the expression of heterochromatic genes differentially from the surrounding repressive heterochromatin, marked by only repressive chromatin signatures

Het TAD organization partitions the pericentromeres into active and inactive Het domains that encompass similarly expressing heterochromatic genes Fig. 7a. In the HP1a or Su(var)3-9 RNAi conditions, this genome organization is disrupted; however, the transcription of heterochromatic genes is minimally affected (Fig. 7b). dADD1 and dMES-4 are important *trans* factors that along with heterochromatic factors (HP1a and Su(var)3-9) are enriched at a subset of active heterochromatic genes (Fig. 7c). These factors plausibly maintain the epigenetic landscape that is distinct from the neighboring heterochromatin, the mechanisms of which are yet to be delineated.

To put things in perspective with the existing findings, we propose the following scenarios. First, in the case of HP1a knockdown condition, the stabilization of dADD1 binding at the heterochromatic genes is reduced due to the presence of only H3K9me3 marks and, therefore, more heterochromatic gene expression gets affected as compared to the Su(var)3-9 knockdown. Second, when Su(var)3-9 is knocked down, other H3K9 methyltransferase can deposit the H3K9me3 required for dADD1 localization. Since dADD1 binds to both HP1a and H3K9me3, heterochromatic gene expression is only weakly affected by knockdown of only one of the two—thereby justifying the observation that only a few genes get affected in HP1a or Su(var)3-9 RNAi conditions. Additionally, there are also heterochromatic genes that are not dependent on dADD1-mediated regulation. Thus, dissecting the interrelationship of dADD1, HP1a, Su(var)3-9 and dMES-4 in heterochromatic gene regulation will unravel how the evolution of drosophilids shaped the epigenomic toolkit of heterochromatin to sustain the expression of these genes in only certain *Drosophila* species.

## Conclusions

Our results suggest that an interplay of the higher order chromatin organization and the epigenetic factors regulate the heterochromatic gene expression for a major subset of heterochromatic genes. There exists more than one pathway of regulation for different classes of heterochromatic genes and we have a limited understanding of the entire repertoire of *trans* factors that mediate this regulation. Our findings provide a global perspective on the contribution of the hierarchical pericentromeric genome organization and heterochromatic factors (HP1a & Su(var)3-9) in modulating the expression of a majority of heterochromatic genes and revisiting the results of the candidate-based genetic studies in the light of modern genomics approaches.

## Materials and methods

### Cell culture

*Drosophila melanogaster* embryo (20–24 h)-derived S2 cell line was maintained in Schneider’s insect media (GIBCO, Gaithersburg MD) with 10% heat-inactivated FBS and 1% Penicillin–Streptomycin, at 25 °C in the tissue culture incubator.

### 3C and 5C library preparation

The 3C and 5C libraries were prepared as described previously in [50].

Generation of 3C control library: Purified genomic DNA from S2 cells was used for generating the 3C control library. 20 µg of genomic DNA was digested at 37 °C overnight using EcoRI (NEB R0101L) restriction enzyme. The digested genomic DNA was purified using phenol:chloroform (1:1vol/vol). The ligation reaction was performed at 16 °C overnight using T4 DNA Ligase (Invitrogen, cat. no. 15224-025). The digested and ligated DNA is the 3C control library and is suspended in 200ul TE.

Generation of the 3C library: 5 × 10^7^ S2 cells were grown to confluence and 95–98% viability in Schneider’s media (*n* = 3 biological replicates). Cells were resuspended in 22.5 ml of PBS and proceeded for crosslinking using 37% formaldehyde (Sigma-Aldrich) for 10 min and 1.25 ml of 2.5 M glycine (5 min room temperature and 15 min on ice) was used for quenching. Crosslinked cells were disrupted on ice using 15 strokes in a Dounce homogenizer (pestle A). The cell lysate was centrifuged for 5 min at 2,000 g at RT and suspended in 500 µl of 1× Restriction buffer 2 (NEB) and divided in 10 aliquots of 50 µl each. 337 µl of 1× restriction buffer and 38 µl of 1% SDS was added to each aliquot, which was incubated at 65 °C for 10 min. 44 µl of TritonX-100 was added to each tube to quench the SDS. 400 U of EcoRI (NEB R0101L) was added and incubated overnight at 37 °C. 86 µl of 10% SDS was added and incubated at 65 °C for 30 min. The ligation reaction was set up as follows: Cell lysate—575 µl; 10X T4 DNA ligase buffer—745 µl; 10% Triton X-100—745 µl, 10 mg/ml BSA (B9001S)—80 µl, 100 mM ATP (Sigma A9187)—80 µl; T4 DNA ligase (NEB M0202L): 10 µl and water—5.96 ml. The ligation was performed at 16 °C for 2 h. 50 µl of 10 mg/ml Proteinase K (Invitrogen, cat. no. 25530-031) was added followed by overnight incubation at 65 °C. DNA was extracted using 8 ml of 1:1 vol/vol phenol:chloroform. To the aqueous phase, 3 M sodium acetate pH 5.2 and chilled absolute ethanol were added to precipitate the 3C DNA. The library was dissolved in TE pH-8 and RNaseA (ThermoFisher EN0531) treatment was performed for 15 min at 37 °C. The 3C library ran as a tight band of size greater than 10 kb, with very little RNA and no undigested genomic DNA in the well. PCR titration was performed using primers from a gene desert region in the *Drosophila melanogaster* genome [51]. Quality control of the 3C library was performed using positive interactions from reported interactions in the literature [52]. The 3C library generated was used for 5C library preparation or for PCR reactions (primers: Additional file 1: Table S3) to validate the 5C interactions (from the sequencing data). 1 K/2 K primers were used as internal controls [53].

### 5C Primer design and dilution

5C primers were designed using my5C (my5C.primers) tools [54] for the pericentromeric regions: chr2L: 21900975–23011544, chr2R: 1–1385689, chr3L: 22855576–24543557, chr3R: 1–428656, chrX: 21600796–22422827. Parameters used in primer design include: U-BLAST, 3; S-BLAST, 50; 15-MER: 800; MIN_FSIZE, 100; MAX_FSIZE, 50,000; OPT_TM, 65; OPT_PSIZE, 30. Primers were excluded wherever unique mapping was not possible with highly repetitive sequences. The universal T7 (5′-TAATACGACTCACTATAGCC-3′) and T3 sequence (5′-TATTAACCCTCACTAAAGGGA-3′) were added to forward and reverse primers, respectively. 5C forward and reverse primers were pooled separately and diluted to the 50 µM final concentration. The reverse primers were phosphorylated using Polynucleotide kinase (NEB M0236S).

### Conversion of 3C libraries to 5C libraries

3C and control libraries were separately mixed with salmon sperm testis DNA (total DNA mass of 1.5 µg). 3C library taken represents approximately 150,000 genome copies to reflect the library complexity. 2.7 µl of cold 5C primer mix containing 1 µl of 10× 5C annealing buffer (NEB B7004S) and 1.7fmol of each 5C primer was added and incubated at 95 °C for 5 min to denature the libraries and the primers, followed by incubating the mix at 48 °C for 16 h to anneal primers to 3C template library. 20 µl 5C ligation buffer containing 10U Taq DNA ligase (NEB M0208S) was added and incubated at 48 °C for 1 h to ligate 5C primers annealed to 3C junctions. The reaction is terminated by incubating at 65 °C for 10 min. No ligase, no template, and no primer controls were included in the PCR reactions set up to amplify the ligated 5C junction using universal T3 and T7 primers. The libraries were run on E-gels (Invitrogen G661012) and purified from there to allow a minimal loss before proceeding for Illumina sequencing library preparation (as per the Illumina manual).

### RNAi-mediated knockdown

For RNAi knockdown of HP1a, Su(var)3-9 and dADD1 proteins, primers were designed at the exons ranging in size from 300 to 1000 bp. For proteins with isoforms, primers (T7 overhangs on both forward and reverse primers) were designed on the common exons to ensure knockdown of all the isoforms. Using the primers, the exonic regions were amplified and gel eluted to use as a template for generating double-stranded RNA using in vitro transcription reactions. Primers against EGFP were used to generate dsRNA for the control (mock RNAi).

### In vitro transcription

1 µg PCR template DNA was used as a template for the in vitro transcription reaction as per the instructions of the Ambion MEGAscript kit (cat no. 1334). The RNA was extracted using phenol/chloroform and precipitated using isopropanol. An aliquot was run on agarose gel to check the integrity of the total RNA and quantified using NanoDrop spectrophotometer.

### dsRNA transfection for RNAi-mediated knockdown

S2 cell transfections were done using Qiagen Effectene Transfection Reagent (Cat No 301425). 10^6^cells/ml were plated in 6-well plates 24 h before transfection. For each well, 1 µg of dsRNA prepared using an in vitro transcription kit was used. The cells were incubated at 25 °C for 3 days for dsRNA-mediated knockdown of the targeted protein. On the 4th day, the knockdown was validated by immune blotting using antibody against HP1a (DHSB C1A9) and Su(var)3-9 (Abcam ab4811) and using qPCR for dADD1 knockdown. Cells transfected with dsRNA against EGFP were used as control. The RNAi experiments were done in duplicates for 5C sequencing and triplicates for total RNA sequencing.

### RNA preparation for RNA seq libraries

S2 cells were harvested and pelleted. 1 ml of TRIzol reagent was added per 10^6^ cells and pipetted to homogenize the cell lysate. RNA precipitated using isopropanol, dissolved in DEPC treated TE buffer and checked for quality before proceeding for library preparation using Illumina RNA seq library preparation kit.

### cDNA preparation

The template RNA was treated with DNaseI (NEB M0303S) 1:100 dilution to destroy any residual DNA during the RNA extraction procedure. The cDNA for reverse-transcriptase PCR is prepared as per the manufacturer’s instruction of PrimeScript™ first strand cDNA Synthesis Kit from TaKaRa (6110A). The cDNA was appropriately diluted to proceed for quantitative PCR using SYBR Green (ThermoFisher 4309155) and ABI quantitative PCR machine. Primers used are listed in Additional file 1: Table S3.

### Chromatin immunoprecipitation

1X10^6^ S2 cells grown to confluence, and 95–98% viability, were used for preparing the chromatin. The cells were fixed using 1% formaldehyde for 10 min at room temperature and quenched using 2.5 M glycine. The cells were lysed in 1× Lysis buffer (1% SDS, 1 mM EDTA and 10 mM Tris pH-8 and protease inhibitors) for 30 min. Shearing of the chromatin (150–200 bp) was done using Pico Bioruptor for 20 cycles—30 s on/off. The chromatin immunoprecipitation was performed as per the manufacturer’s instructions in Diagenode Low Cell ChIP kit. 5–10 µg of chromatin and 1–3 µg H3K36me3 (Abcam ab9050) antibody was used for each reaction. The ChIP-ed DNA was used for qPCR using SYBR Green Master Mix on ABI quantitative PCR machine.

### 5C data analysis and peak calling

5C sequencing was done in triplicates and mapped to the dm3/R5 genome build of *Drosophila melanogaster*. For all the libraries, because of common ends of all 5C primers, trimming of both 5′ (1–20 bases) and 3′ (80–100) ends was done with fastx_clipper from the fastx_toolkit. Reads of poor quality were removed using fastq_quality_filter. Only reads with a minimum Phred score of 25 across 80% of the reads were retained. Reads passing the quality filters were mapped to a custom reference genome made from all possible combinations of forward/reverse 5C primers using bowtie2. Reads that mapped to multiple combinations were discarded. Interaction frequency list (total number of reads aligning to a primer pair in the 5C samples to the control (irrespective of whether both or only one of the paired-end reads mapped to the pair) were generated using a custom Perl script, and the frequencies were normalized to the total mapped read count. The normalized lists were further normalized to expected frequency using the HiTC package in BioConductor R [55]. The normalization factor used was 11.62 for control and 8.65 for 5C replicates. The resulting data were saved as pairwise-interaction lists for each sample. These pairwise interaction lists were uploaded to the my5C tool (http://my5c.umassmed.edu), developed by Dekker lab) [54], Parameters used in my5C.heatmap module: Experiment 1: Control (non-crosslinked) sample interaction list, Experiment 2:5C experiment interaction list. In obs-exp tab Interaction frequency scores are normalized for distance by dividing the observed value by the expected value and log2 obs/exp ratio is > 0, it means that the interaction frequency between the two fragments is higher than expected based on distance and, therefore, a valid interaction. Interaction frequency scores are plotted as heatmaps, using Binning parameters: Bin size 10 kb (5 kb for Chr X), Bin step 1 kb, Binning mode median, B-0’s-checked and smoothing parameters—smoothing type: by interactions, S X/Yaxis = 10,000, S mode = median and S-0’s checked. The pairwise interaction score files for each chromosome arm and replicate are downloaded as pairwise interaction files for visualization in genome browsers and TAD calling. For generation of arc diagrams of the 5C interactions, the pairwise-interaction files obtained from my5C were uploaded into WashU Epigenome Browser [56] to visualize the interactions along with tracks of RefSeq genes, ChIP data of chromatin marks, RNA Pol II and replication timing data. The plots only show interactions with a positive interaction score (~ enriched over the (non-crosslinked) control).

### Domain calling using Directionality Index and HMM

The read normalized 10 kb binned 5C pairwise interaction files for each chromosome were saved from my5C web-tool. TAD boundaries were obtained using the Directionality Index method described in [28, 29] and available at http://doc.genomegitar.org/DI_calculation.html. This method is based on the concept that regions at the periphery of TAD are highly biased in their interaction frequencies. It is based on Chi squared test statistic where the null hypothesis is that bins do not show biased upstream and downstream interaction. Directionality Index (DI) calculation was performed using an R script, in a 100 kb × 100 kb square along the diagonal of the interaction frequency matrix for each replicate using the following formula:

$$DI \, = \, \left( {B - A/|B - A|} \right) \, \left( {\left( {A - E} \right)^{2} /E \, + \, \left( {B - E} \right)^{2} /E} \right),$$

where *A* is the sum of all interactions from a given 10 kb bin to the upstream till 100 kb, *B* is the sum of all interactions from a given 10 kb bin to the downstream till 100 kb, *E* is the expected number of interactions for each bin under the null hypothesis and it equals (*A* + *B*)/2.

To predict TADs after the estimation of DI, a Hidden Markov Model was constructed. The MATLAB script for HMM previously described in [29] was rewritten in R language using CRAN package HMM. TADs were predicted across the replicates for each chromosome except for chr3R (because of too few interactions mapped, the HMM did not pick up any signals). TAD boundary called for each replicate was pooled for a consensus boundary definition. Thus, to identify consensus TAD borders across replicates, a combined distribution plot for the distance between boundaries called for three replicates was plotted. Boundaries within 50 kb of each other among replicates were considered consistent (> 72.3% of boundaries called across chromosomes overlapped within this window). Using these parameters, consensus TADs and TAD borders were defined for each chromosome.

### Feature overlaps—intra-chromosomal and inter-chromosomal interaction datasets

The BEDTools suite was used to compute overlap of various genomic and epigenomic features with the intra-TAD and TAD boundaries [57]. BED files for each chromosome (intra-chromosomal interactions) were binned into 10 kb windows (5 kb for chrX). Scaled TAD border of 30 kb (with 10 kb bin) and scaled intra-TAD region of 150 kb (with 30 kb) as per the median size of TAD borders and intra-TAD regions was considered. The number/frequency of overlap of each genomic feature with each 10 kb/30 kb bin was calculated to generate a matrix that was subsequently used for the heatmap. The results were plotted as a heat map for each chromosome with features on the *y*-axis and 10 kb/30 kb bins of genomic coordinates on the *x*-axis using the ggplot2 package in R. To check for any biases, we went 500 kb upstream/downstream on chromosome arms L and R, respectively.

To calculate the statistical significance of our computed overlaps, OverlapPerm function from regioneRpackage (DOI: 10.18129/b9.bioc.regioner, https://www.bioconductor.org/packages/devel/bioc/vignettes/regioneR/inst/doc/regioneR.html) was used, calculating the p and Z value for the numbers of overlaps of a particular feature with TAD borders as opposed to the randomized dm3 genomic regions. The feature files used were obtained from modENCODE/respective publications as listed in Additional file 1: Table S5 [58].

Similarly, for trans or inter-chromosomal interactions, a BED file was prepared to include all the unique anchor points, 10 kb binned, across the two replicates to represent inter-chromosomal interaction. This was then intersected with various genomic features, repeats, boundary elements and histone modifications as done for intra-chromosomal interactions. The overlaps of features in different combinations were visualized as an UpSet plot [59].

### Comparison of inter and intra-TAD interactions in RNAi conditions

The read normalized pairwise-interaction matrices were obtained from my5C web-tool for HP1a/Su(var)3-9 RNAi 5C datasets, as described in previous sections. A grouped box plot showing inter-TAD and intra-TAD interaction frequency was plotted. For this purpose, the genomic loci defined as TAD from our analysis, for each chromosome, were considered to calculate inter-TAD and intra-TAD interactions. The replicates (*n* = 2 for knockdown samples and *n* = 3 for WT) were pooled while making final inter- and intra-TAD interaction tables by taking a mean value of interaction scores.

### Transcriptome analysis

Gene expression was quantified using paired-end (read length-151 bp) RNA-Seq data for Control, HP1 and Su(var) knockdown samples in triplicates. A preliminary quality check on data for finding errors in library preparation or sequencing was done using FastQC (version 0.11.5). The adapter removal was done using Cutadapt (version 1.11) in a paired-end mode with Phred score cut-off as 30 for both 3′ and 5′ ends and minimum read length of 50 bases [60]. 82–90% reads passed this quality cut-off. The reads were then aligned to the reference genome, i.e., *Drosophila melanogaster* (build dm3) using STAR Aligner (version 2.4.5a) using two-pass mode [61]. The Pearson correlation coefficient (PCC) showed that replicates correlate well among themselves for each condition (r > 0.96 for all, cut-off—> 0.90). The quantification of gene expression into FPKM (Fragments per kilobase per million) and TPM (transcripts per million) followed by differential expression was done using RSEM (RNA-Seq by Expectation–Maximization version 1.3.0) [62].

To see if genes falling in the same TAD follow a similar expression profile, we took the quantification step output files of the RSEM method. From it, FPKM values across replicates for each condition were made into a single table and genes falling within our experimental region were a subset for all further analysis. A mean FPKM value (from the three replicates of RNA seq data) was calculated for each gene and categorized into the following gene expression subgroups: (a) no-expression-0 FPKM (b) low expression 1–10 (c) medium expression 11–50 (d) high expressio*n* > 51. The differential gene expression analysis for KD samples against a control sample with FDR (false discovery rate) cut-off of 0.05 was used to retrieve differentially expressed genes.

This criterion was also used for comparing gene expression with dADD1, H3K36me3, H3K9me3 and HP1a ChIP data. The average profile for genes falling in: no-expression, low-expression, medium-expression, and high-expression category were plotted for along with ChIP data using deepTools (computeMatrix, scale-region and plotProfile packages) [63].

### dMES-4 knockdown analysis

RNA sequencing for knockdown of histone methyltransferase dMES-4 and its appropriate control was taken [64]. The alignment using STAR aligner, differential expression analysis with FDR cut-off (0.05) using RSEM was done as mentioned above. The DEGs falling in our region of the study were shortlisted from this set.

### Replication timing data

The replication timing dataset for *Drosophila melanogaster* (build dm3) was taken from http://www.replicationdomain.com/ [65]. This was overlapped and plotted as an area plot over the identified TADs for our study with replication timing on the y-axis and genomic coordinates on the *x*-axis and visualized graphically.

## Supplementary information

**Additional file 1: Fig. S1**: Heterochromatic regions lack sufficient information about the pericentromeric genome organization, **Fig. S2**: Quality controls for 3C and 5C libraries, **Fig. S3**: Directionality Index plots, **Fig. S4**:Validation of 5C interactions, **Fig. S5**: 5C interaction maps, **Fig. S6**: Inter-chromosomal interactions within the Het Regions, **Fig. S7**: Overlap permutation statistical test for overlap of Het TAD borders with binding sites of various architectural/insulator proteins, **Fig. S8**: Overlap of Het TAD borders with genomic features, **Fig. S9**: Het TADs and repeats, **Fig. S10**: Overlaps of Het TADs with the replication timing domains in the pericentromeric regions, **Fig S11**: Overlaps of Het TADs with the RNA Pol II ChIP peaks in the pericentromeric regions, **Fig S12**: Correlation of Het TAD with expression levels of heterochromatic genes, **Fig S13** RNAi mediated depletion of HP1a or Su(var)3-9 and effects on genome organization, **Fig. S14**: Effects of RNAi mediated depletion of HP1a or Su(var)3-9 on genome-wide transcriptome, **Table** **1**: List of TAD and TAD boundary coordinates, **Table** **2a**: List of differentially expressed genes in HP1a or Su(var)3-9 RNAi conditions, **Table** **2b**: Average FPKM values of H3K9 methyltransferases in the RNAi conditions, **Table** **3**: List of primers, **Table** **4**: List of antibodies, **Table** **5**: List of Next Generation Sequencing datasets of S2 cell line used from NCBI GEO database or modENCODE.

**Additional file 2.** Datasheet for list of 5C primers used in this study.

**Additional file 3.** Datasheet for sequencing statistics of 5C and RNA sequencing datasets.

**Additional file 4.** Datasheet for list of S2 Het MARs used in the study.

**Additional file 5.** Datasheet for list of differentially expressed genes in HP1a KD and Su(var)3-9 KDDatasheet for list of differentially expressed genes in HP1a KD and Su(var)3-9 KD.

**Additional file 6.** Datasheet for list of differentially expressed genes in dMES-4 KD.

## Data Availability

Genomic datasets are deposited in the NCBI GEO database with accession number GSE126952. In-house R scripts used in the analyses are available upon request.
