# Supplementary material for: Interplay of pericentromeric genome organization and chromatin landscape regulates the expression of Drosophila melanogaster heterochromatic genes
Source: Epigenetics Chromatin. 2020 Oct 7;13:41. doi: 10.1186/s13072-020-00358-4 (PMC7541242; doi:10.1186/s13072-020-00358-4)
Supplement: Supplementary file 1 — Additional file 1: Fig. S1: Heterochromatic regions lack sufficient information about the pericentromeric genome organization, Fig. S2: Quality controls for 3C and 5C libraries, Fig. S3: Directionality Index plots, Fig. S4:Validation of 5C interactions, Fig. S5: 5C interaction maps, Fig. S6: Inter-chromosomal interactions within the Het Regions, Fig. S7: Overlap permutation statistical test for overlap of Het TAD borders with binding sites of various architectural/insulator proteins, Fig. S8: Overlap of Het TAD borders with genomic features, Fig. S9: Het TADs and repeats, Fig. S10: Overlaps of Het TADs with the replication timing domains in the pericentromeric regions, Fig S11: Overlaps of Het TADs with the RNA Pol II ChIP peaks in the pericentromeric regions, Fig S12: Correlation of Het TAD with expression levels of heterochromatic genes, Fig S13 RNAi mediated depletion of HP1a or Su(var)3-9 and effects on genome organization, Fig. S14: Effects of RNAi mediated depletion of HP1a or Su(var)3-9 on genome-wide transcriptome, Table 1: List of TAD and TAD boundary coordinates, Table 2a: List of differentially expressed genes in HP1a or Su(var)3-9 RNAi conditions, Table 2b: Average FPKM values of H3K9 methyltransferases in the RNAi conditions, Table 3: List of primers, Table 4: List of antibodies, Table 5: List of Next Generation Sequencing datasets of S2 cell line used from NCBI GEO database or modENCODE. [file 13072_2020_358_MOESM1_ESM.pdf]

Additional File 1: Supplementary Information

Interplay of pericentromeric genome organization and chromatin landscape regulates the expression of *Drosophila melanogaster* heterochromatic genes

Parna Saha[1,2], Divya Tej Sowpati[1], Mamilla Soujanya[1,2] , Ishanee Srivastava[1] and Rakesh Kumar Mishra*[1,2]
[1]CSIR- Centre for Cellular and Molecular Biology, Hyderabad-500007, India
[2]Academy of Scientific and Innovative Research (AcSIR), India

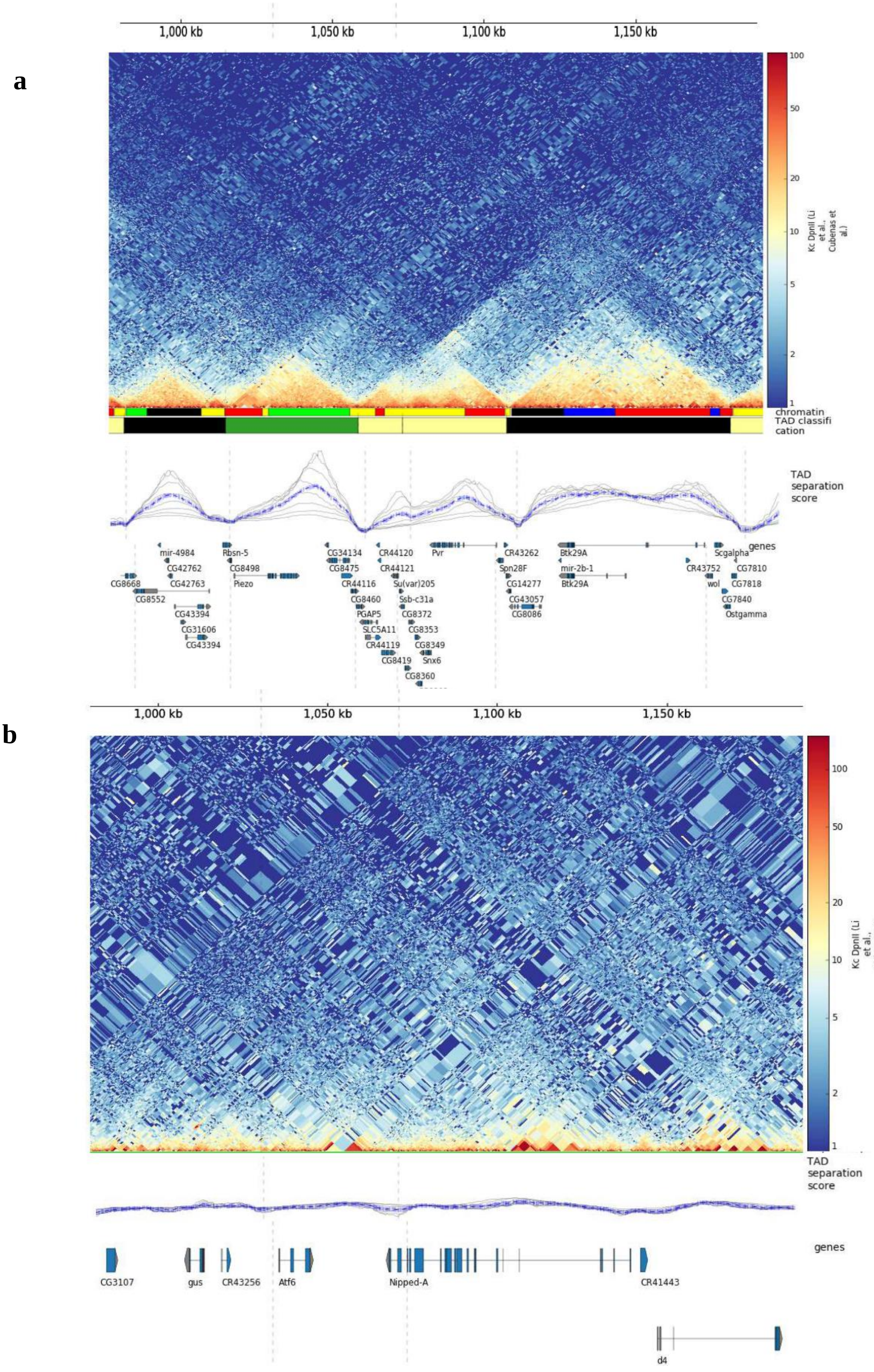

Fig S1: Heterochromatic regions lack sufficient information about the pericentromeric genome organization- a) Snapshot of an euchromatic gene, Su(var)205 gene (chr2L: 8126001- 8310427), showing the TAD structure (Hi-C data for Kc167 cells [Li *et al* & Cubentas *et al*], followed by the TAD separation score, which is inversely correlated with the insulation index between adjacent TADs. **b)** Snapshot for a heterochromatic gene, Nipped A (chr2R:982668-1188470) shows limited information about the TAD structure, in the publicly available dataset. The figures are generated using Chorogenome Navigator (http://chorogenome.ie-freiburg.mpg.de).

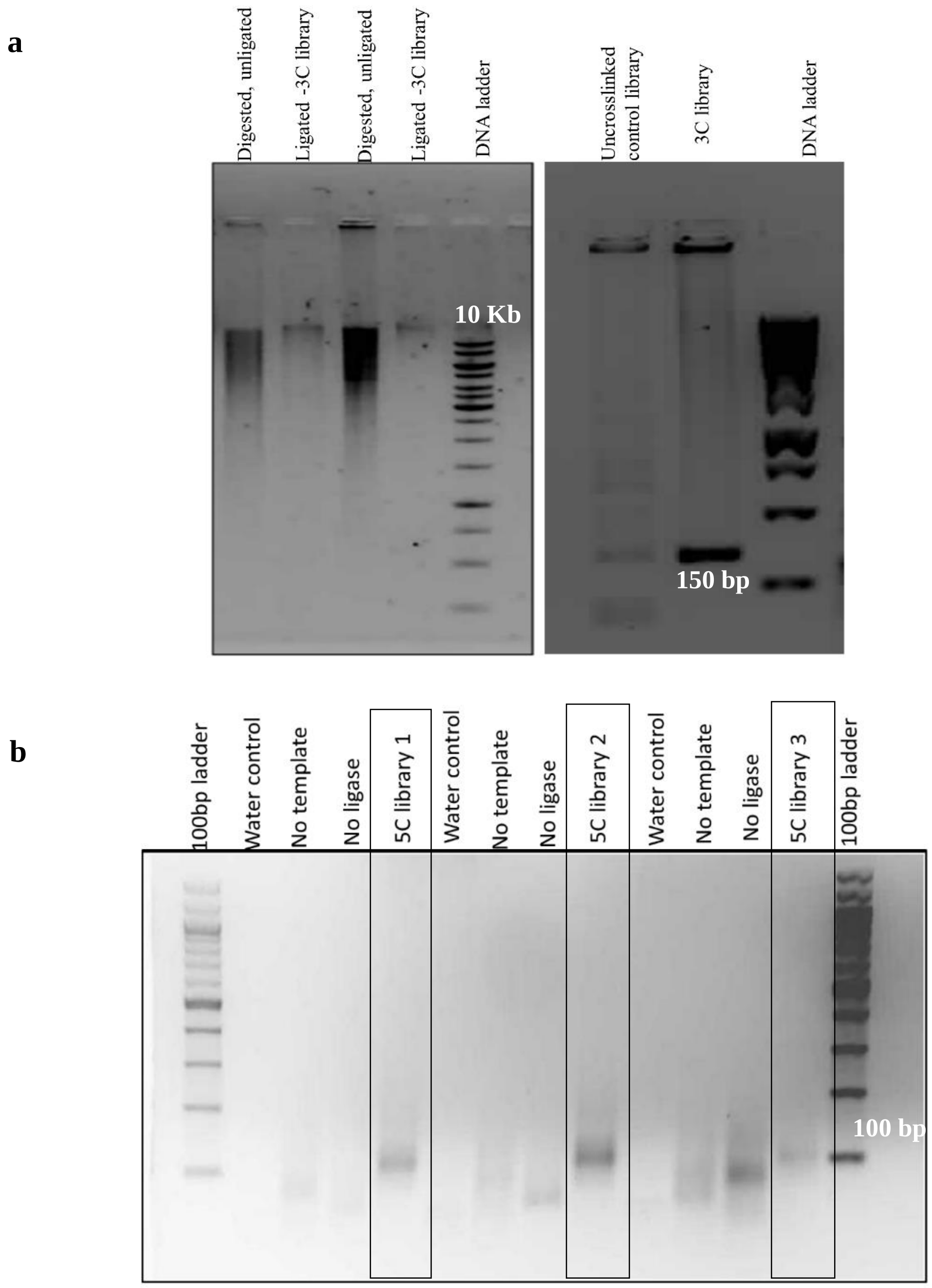

Fig S2: Quality controls for 3C and 5C libraries a) Agarose gel images showing the 3C chromatin which runs as a smear but retracts to a tight band that runs above 10kb upon successful ligation. Second gel image- PCR with positive control primers (Li, H.B *et al*, PLoS Genet. 2013), the control 3C library gives multiple bands as opposed to the single prominent product of 150 bp for the 3C library. This indicates that the 3C library preparation is good and has the representation of the already reported interactions. **b)** Agarose gel image of the 5C PCR reactions of 3C template with no template, no ligase, no primer controls. The 5C library is 100bp in size as the primers are 50 bp each (30 bp complementary to sequence adjacent to the EcoRI sites, including half of the EcoRI site and 20 bp for the T7/3 overhangs).

chr2L

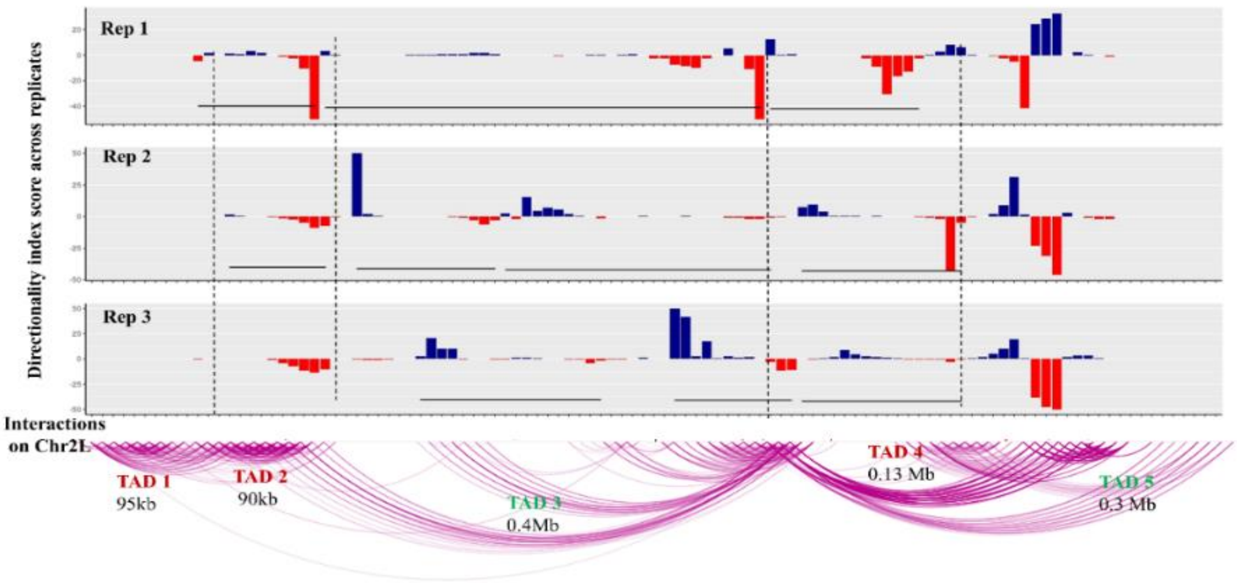

chr2R

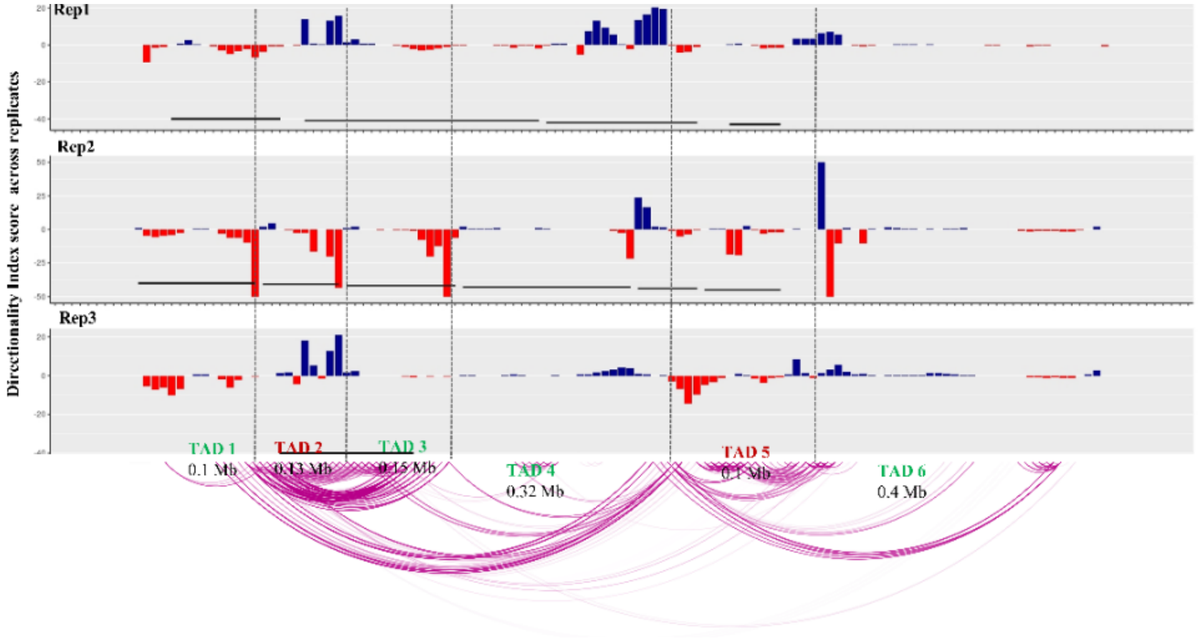

Fig S3: Directionality index plots- for chr 2L, 2R, 3L and X, used to call the consensus TADs across 3 replicates**.**

chr3L

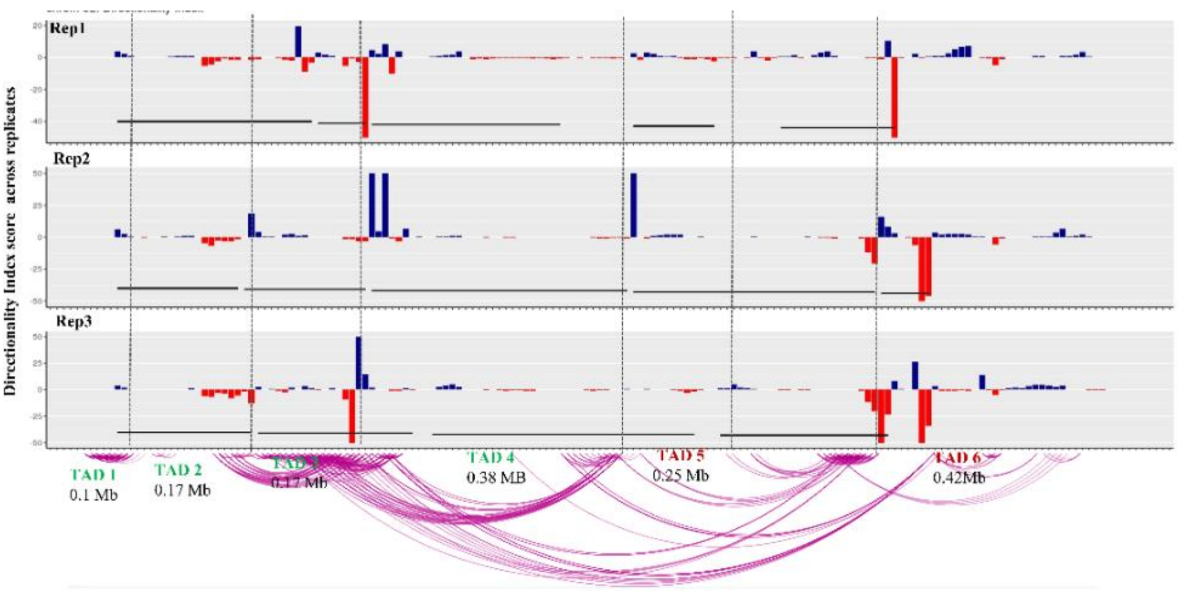

chr X

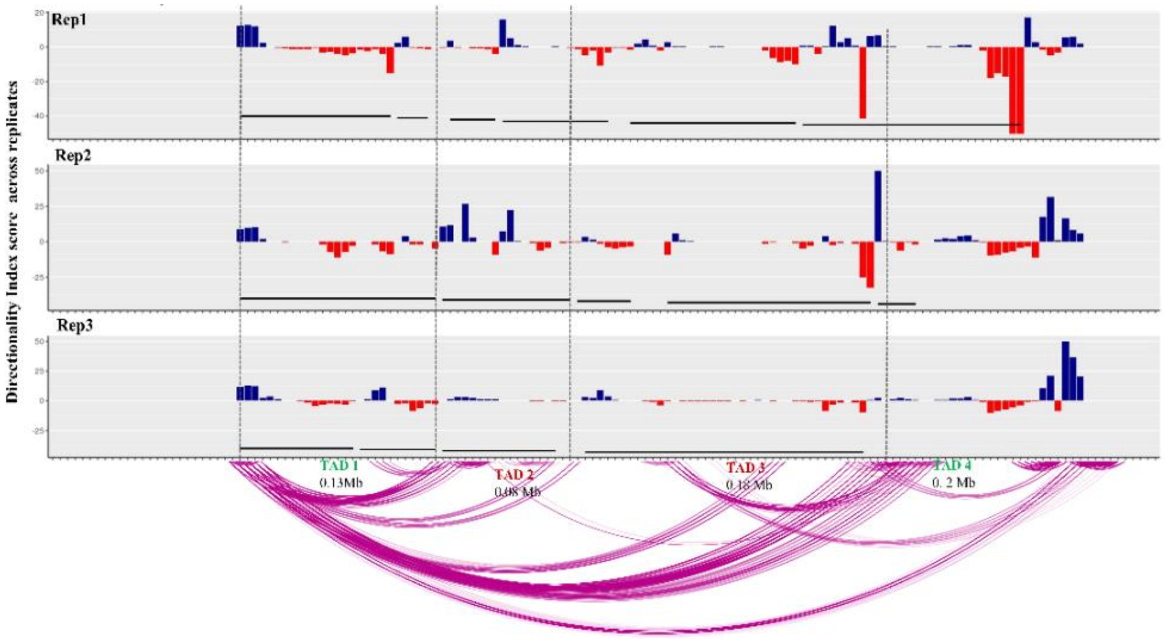

Fig S3: Directionality index plots- for chr 2L, 2R, 3L and X, used to call the consensus TADs across 3 replicates**.**

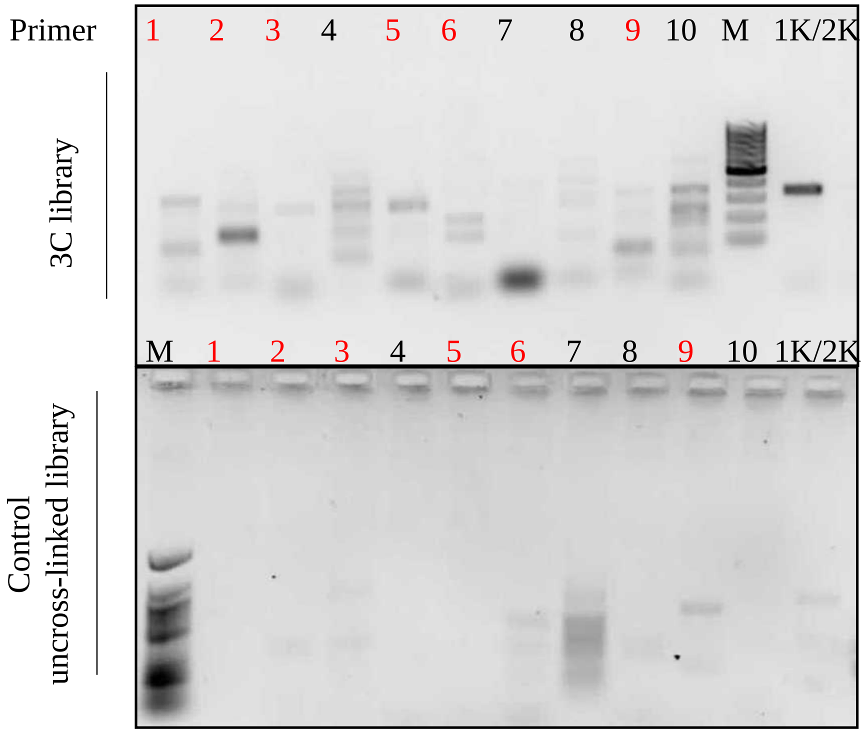

Fig S4: Validation of 5C seq interaction- Agarose gel image showing the validation of candidate 5C interactions reported by 5C seq across chromosome arms using 3C library (has amplification) and control un-crosslinked library (no amplification). 1K/2K are positive control primers -pointing in same direction and adjacent EcoRI sites near *Brk* gene in euchromatin that can only amplify from crosslinked 3C junctions (taken from Li HB *et al*; MCB, 2011). For primers, see Table 3. Each interaction is denoted by a primer pair and the corresponding number of the forward and reverse primer pair number from the 5C primer pool is included. Primers pairs in red are the validated examples.

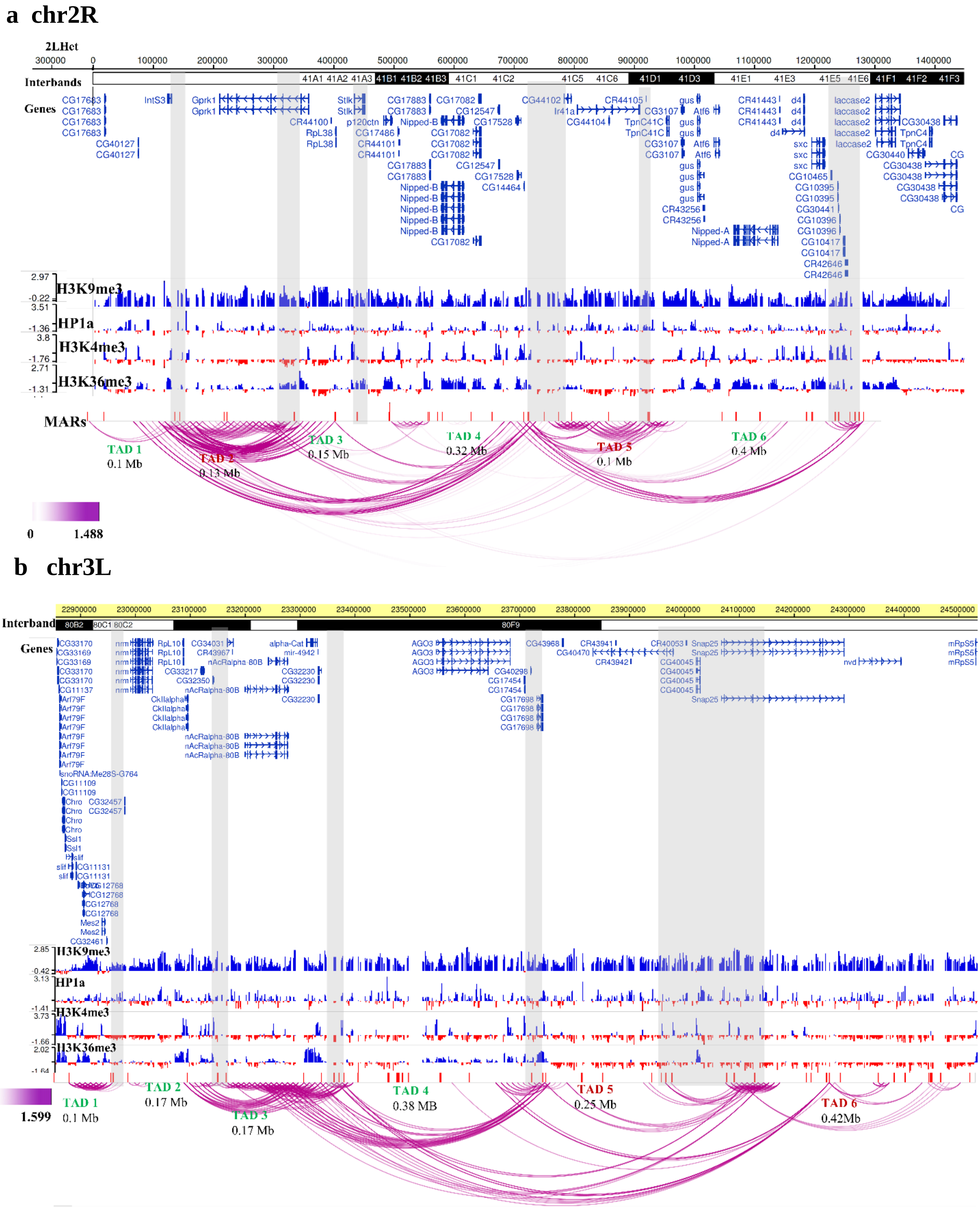

Fig S5: 5C interaction maps- showing the domains and subdomains mapped the pericentromeric regions of **a)** chr2R **b)** chr3L along with the Refseq genes, distributions of H3K9me3, HP1a on heterochromatic genes and H3K4me3 and H3K36me3 marks to indicate the epigenetic state of the domains. TAD colored green and red to indicate active and repressed TADs respectively and sizes are indicated. MARs in red vertical bars and TAD borders are indicated by grey bars. See Table 1 for the coordinates of TADs and TAD borders.

c chrX

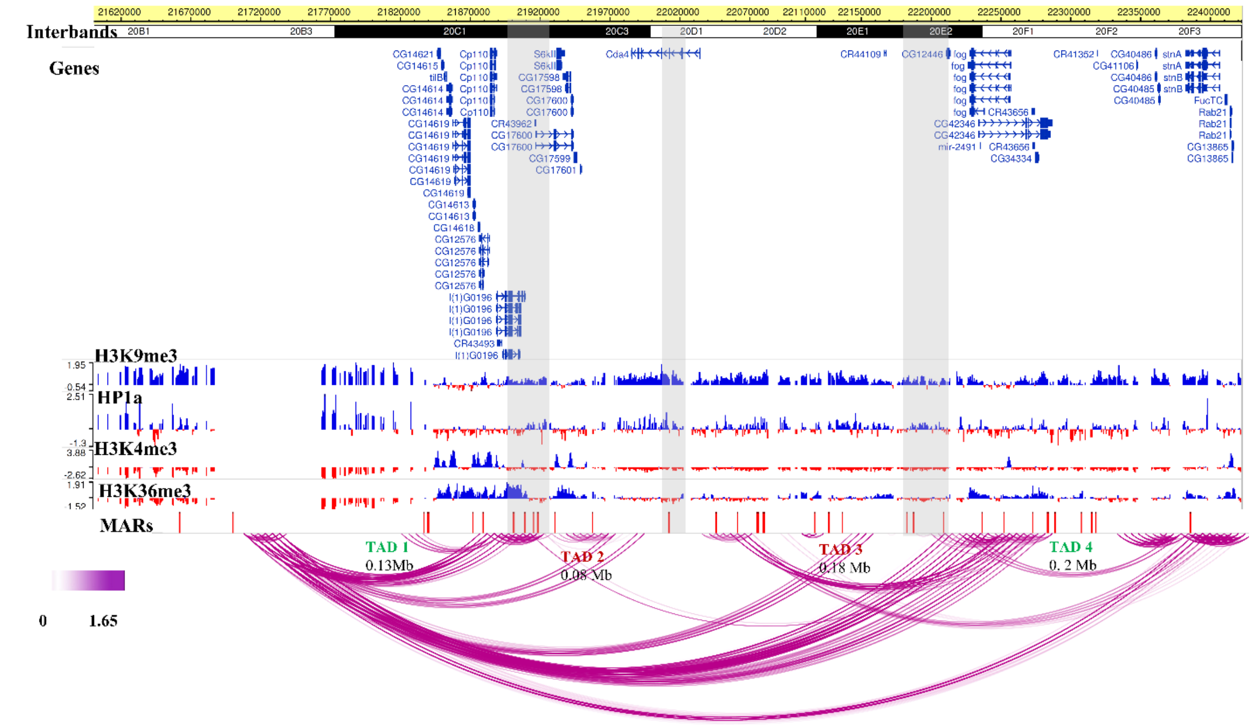

d chr3R

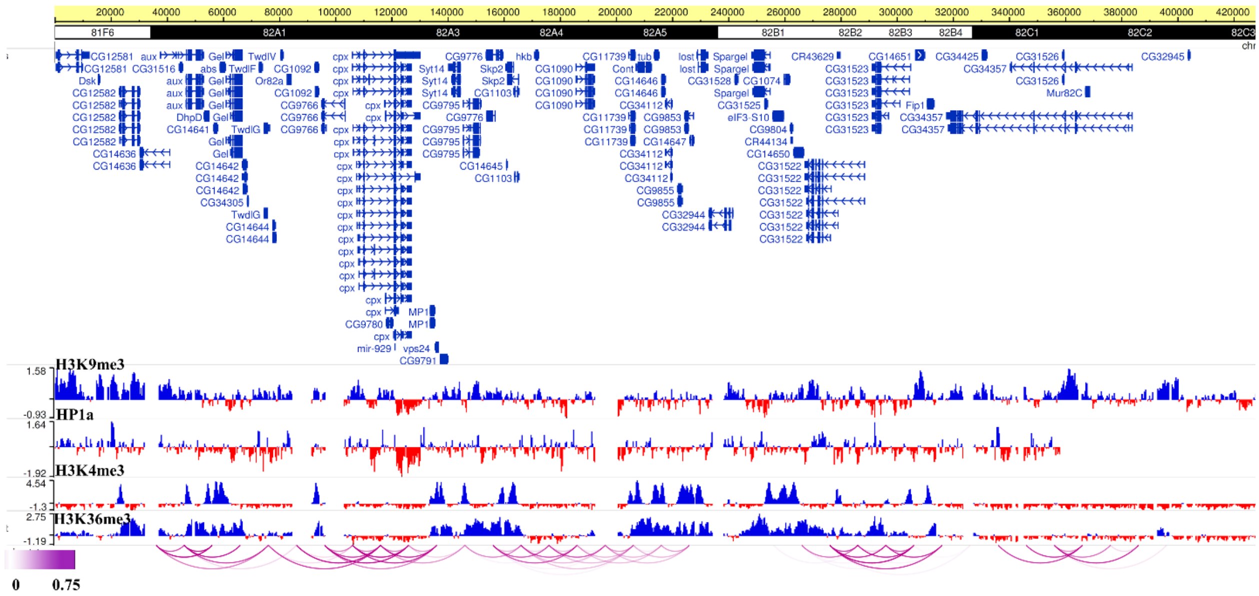

Fig S5: 5C interaction maps- showing the domains and subdomains mapped the pericentromeric regions of **c)** chrX **d)** chr3R along with the Refseq genes, distributions of H3K9me3, HP1a on heterochromatic genes and H3K4me3 and H3K36me3 marks to indicate the epigenetic state of the domains. TAD colored green and red to indicate active and repressed TADs respectively and sizes are indicated. TAD borders are indicated by grey bars. See Table 1 for the coordinates of TADs and TAD borders.

a

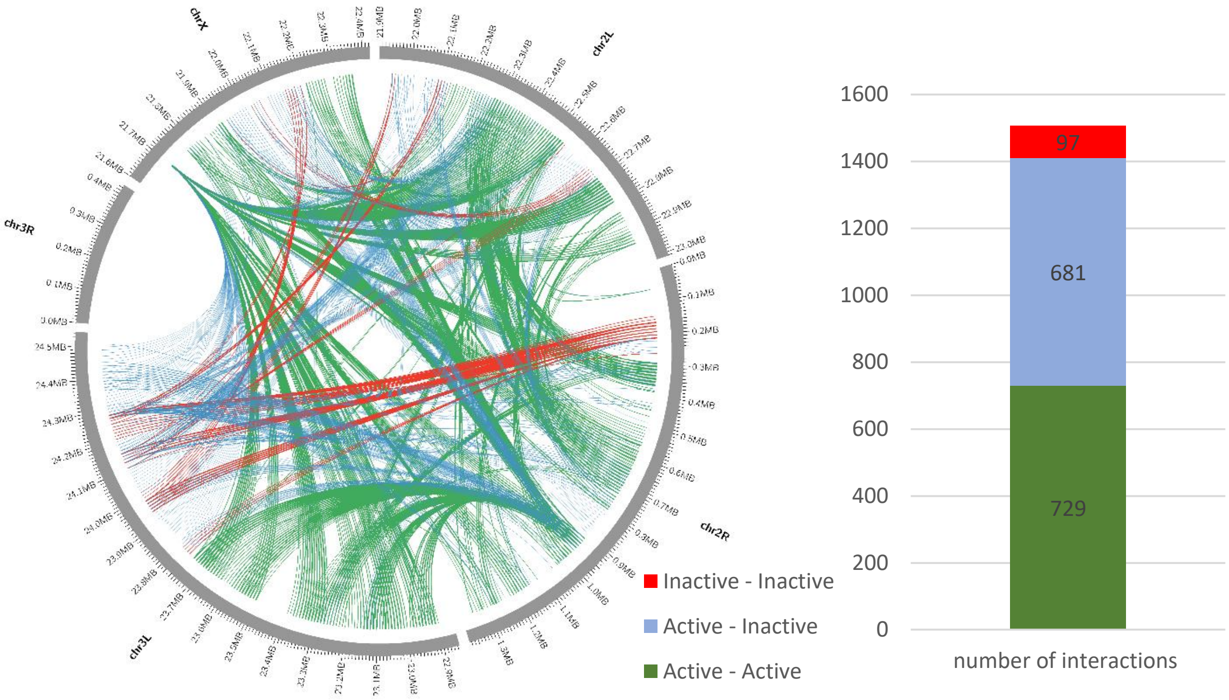

b

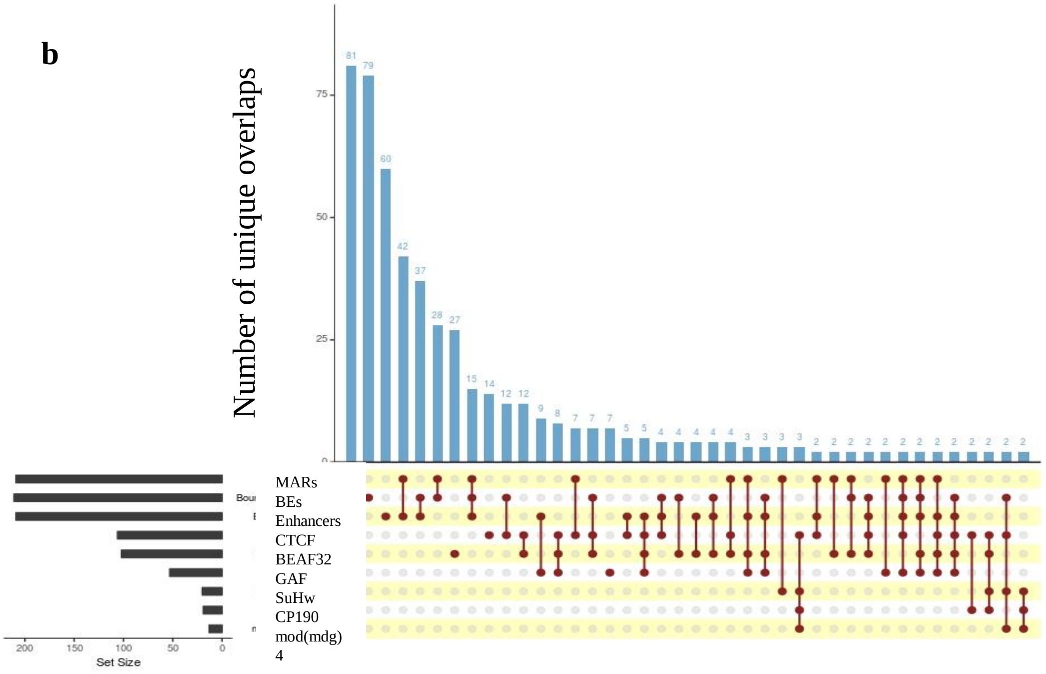

Fig S6: Inter-chromosomal interactions within the Het Regions- a) Circos plot showing the distribution of inter-chromosomal interactions across active and inactive Het TADs mapped on the chromosome arms. **b)** Overlap of inter-chromosomal interacting regions with various genomic features. Number of overlaps indicated on top of the blue bars and number of genomic features overlapped shown in black bars. Red connected dots show the combination of genomic features that overlap with any interacting regions.

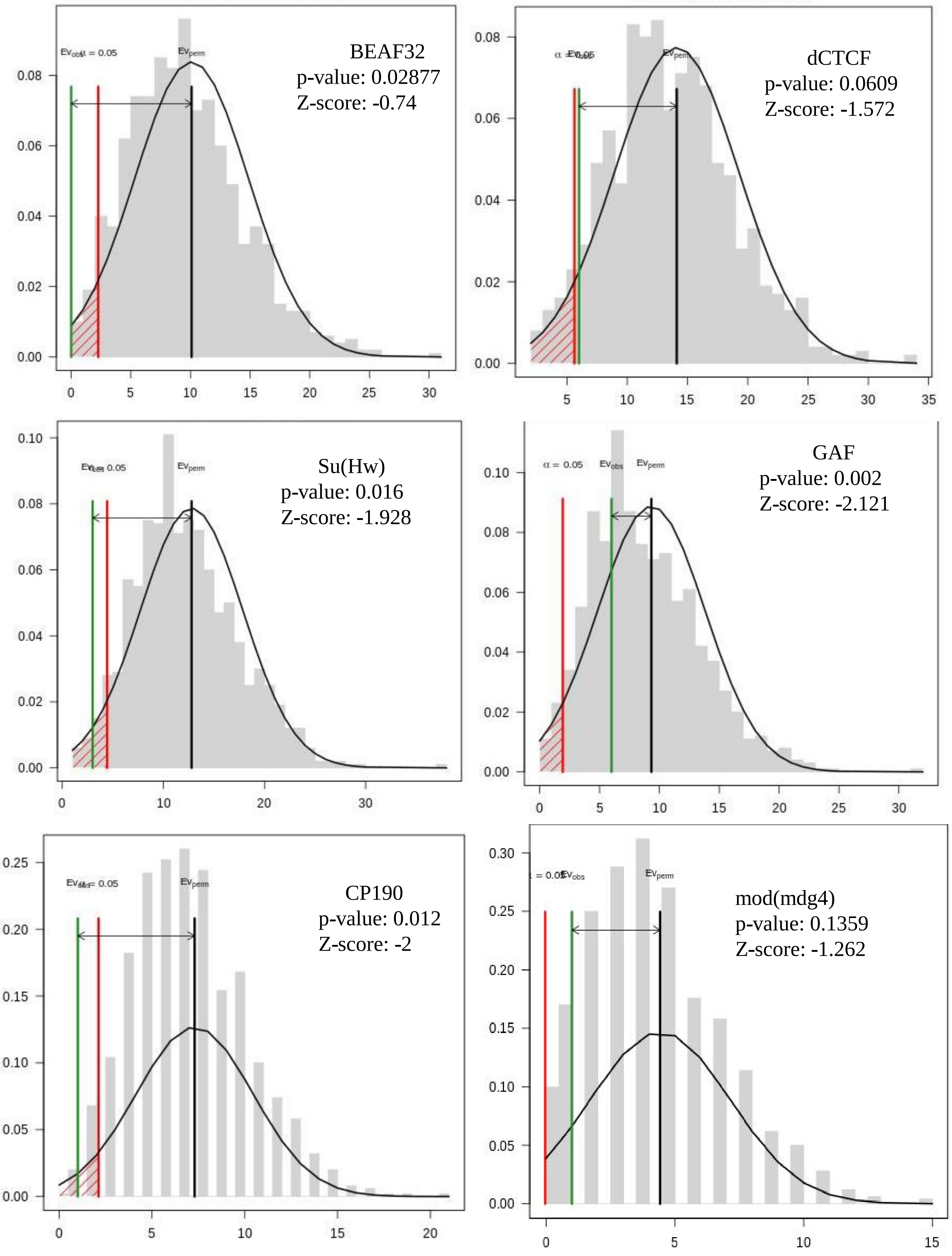

Fig S7: Overlap permutation statistical test for overlap of Het TAD borders with binding sites of various architectural/ insulator proteins – Higher significance of overlap is assigned by low p-value and high Z-score. BEAF32 is enriched at the TAD borders while GAF and mod(mdg4) at the intra TAD regions. dCTCF, CP190 and Su(Hw) are present at the TAD borders but comparable to intra TAD regions. See Fig 2A for the comparative distribution across TADs and TAD borders. n perm = 1000; Y axis – number of overlaps.

a

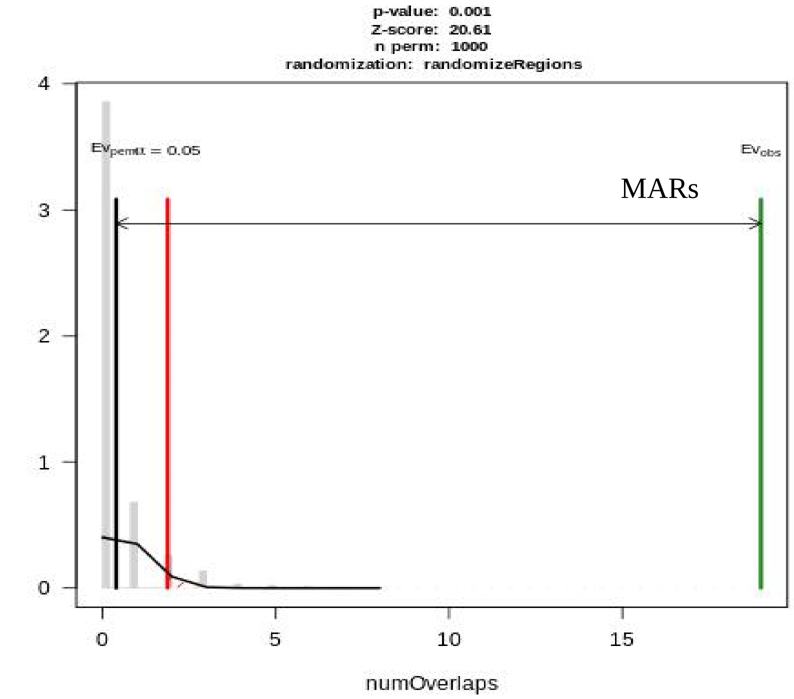

b

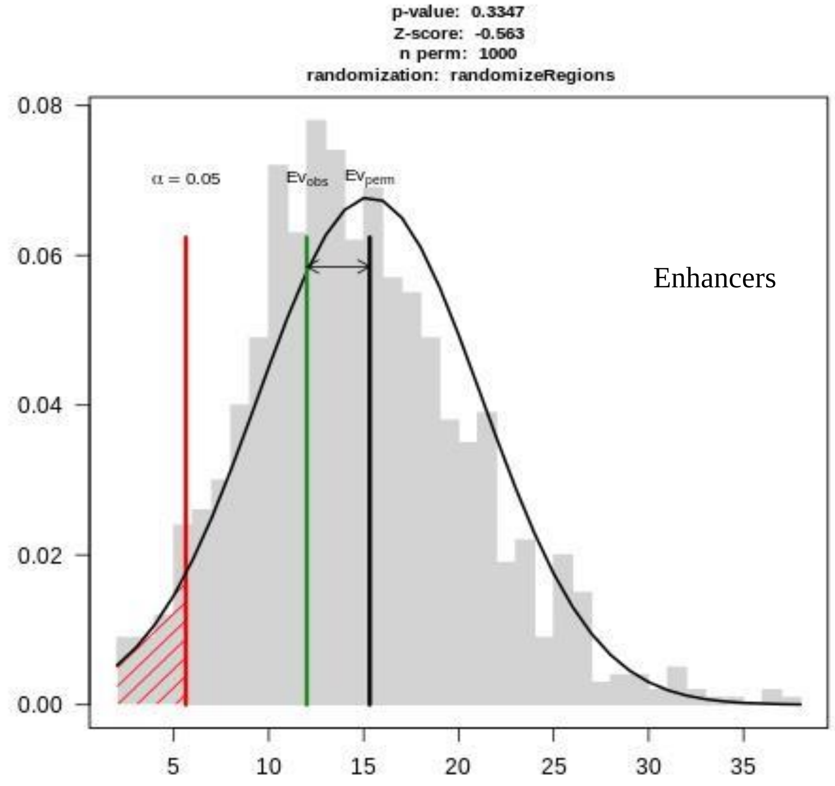

Fig S8: Overlap Het TAD borders with genomic features- Overlap permutation test showing the overlap of TAD borders/inter-TAD regions with various features. In this pictorial representation, the gray histogram represents the number of overlaps of randomized set of regions with the sequence feature (MARs or enhancers in this case) and the black bar denotes the mean. In green is the overlap of the sequence feature with the test regions (TAD borders), which if larger than the randomized regions, the overlap with the test regions is significant. Red bar denotes the significate limit set at $P< 0.05$. z-score measures the strength of the overlap irrespective of the number of permutations. High z-score and low p value indicates significant association. **a)** Overlap permutation test showing the overlap of TAD borders/inter-TAD regions the MARs is highly significant as compared to the **b)** STARR seq enhancers ($p = 0.3347$) indicating promoter- enhancer interactions are likely to encompassed within the TADs

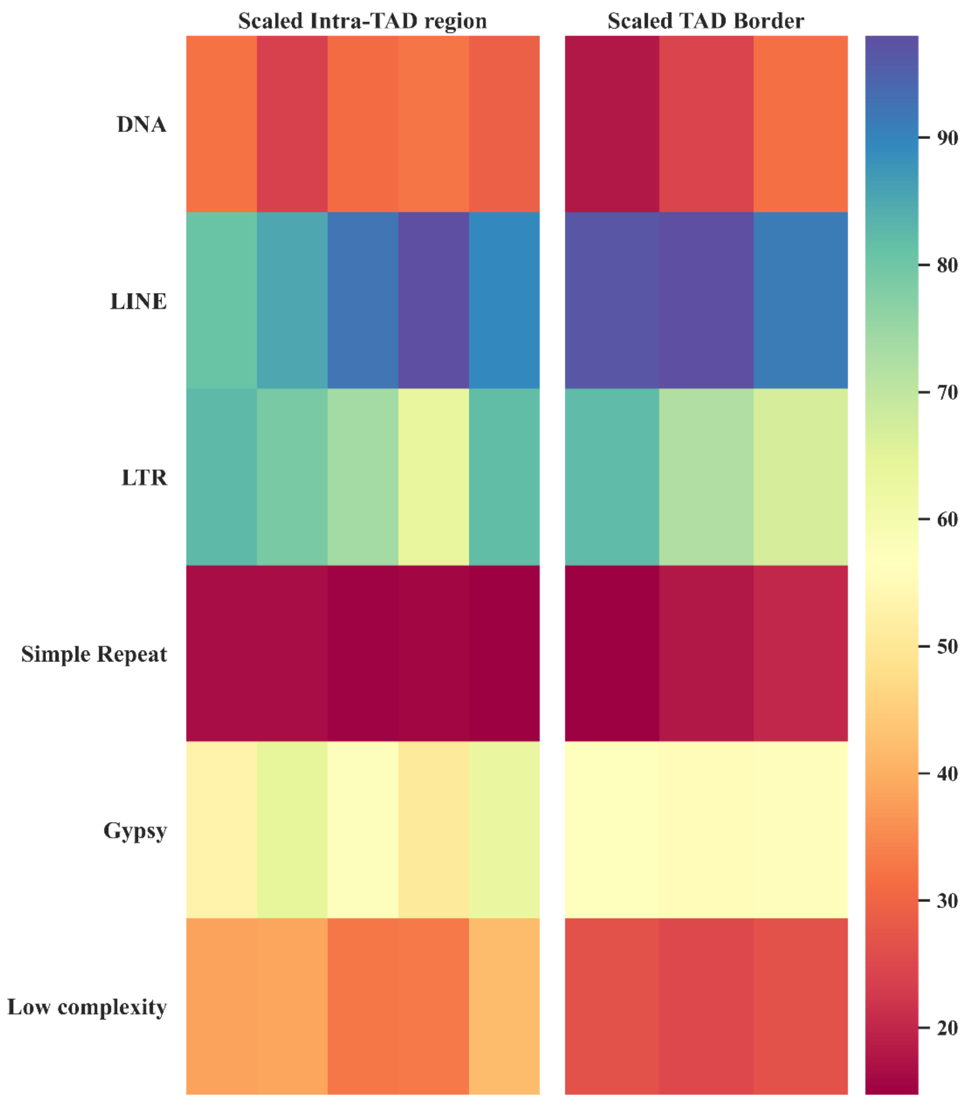

Fig S9: Het TADs and repeats: Overlap of the intra –TAD and TAD border regions with various repeat elements- shows comparable enrichment at the intra TAD regions and TAD borders.

a chr2R

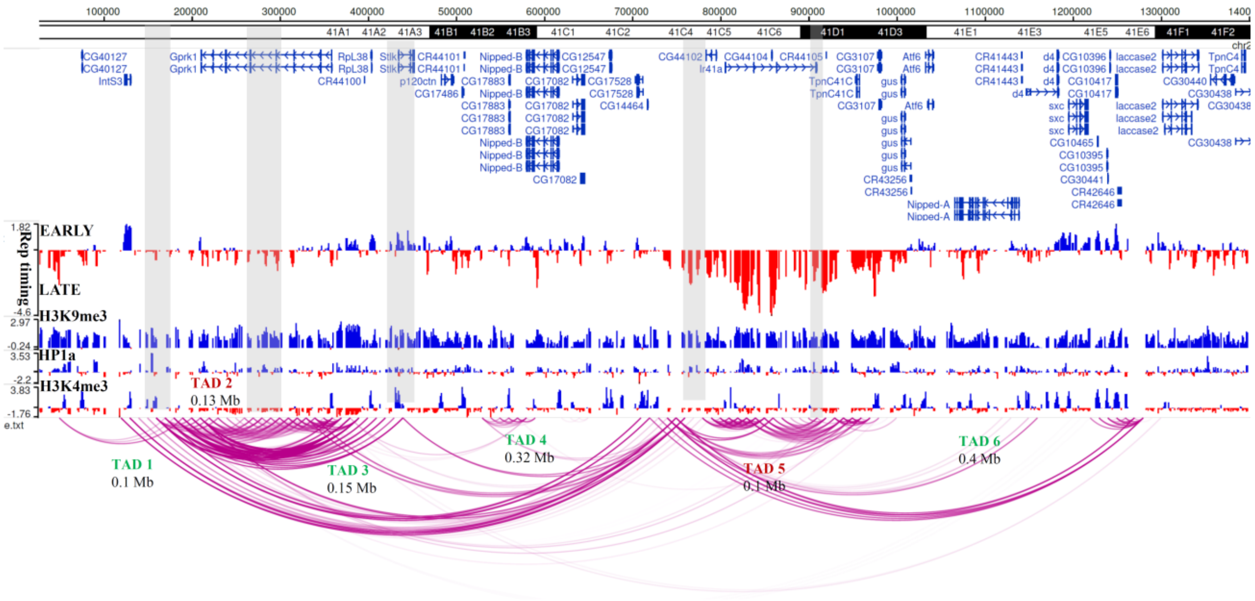

b chr3L

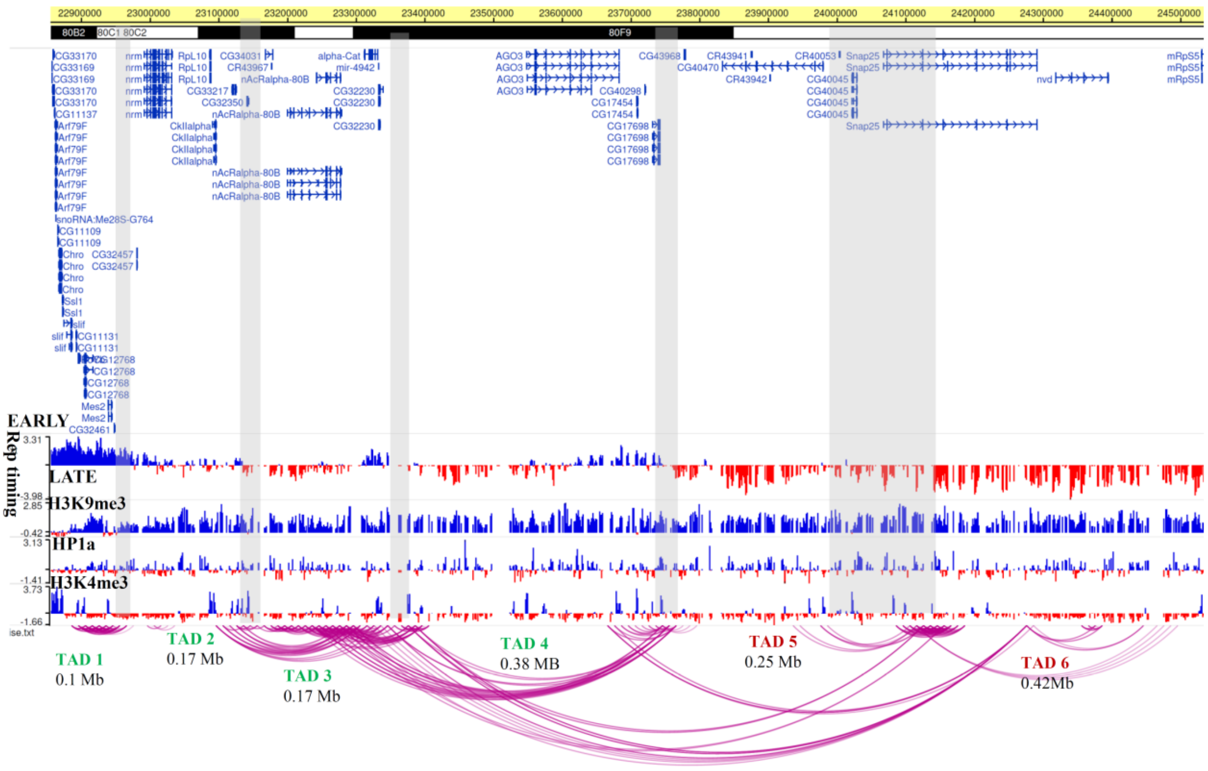

Fig S10: **Overlaps of Het TADs with the replication timing domains in the pericentromeric regions-** Active Het TADs are generally enriched for early replicating regions. Overlay of the replication timing data with long-range interactions in **a)** **c**hr2R **b)** **c**hr3L. Grey bars indicate TAD borders.

c chrX

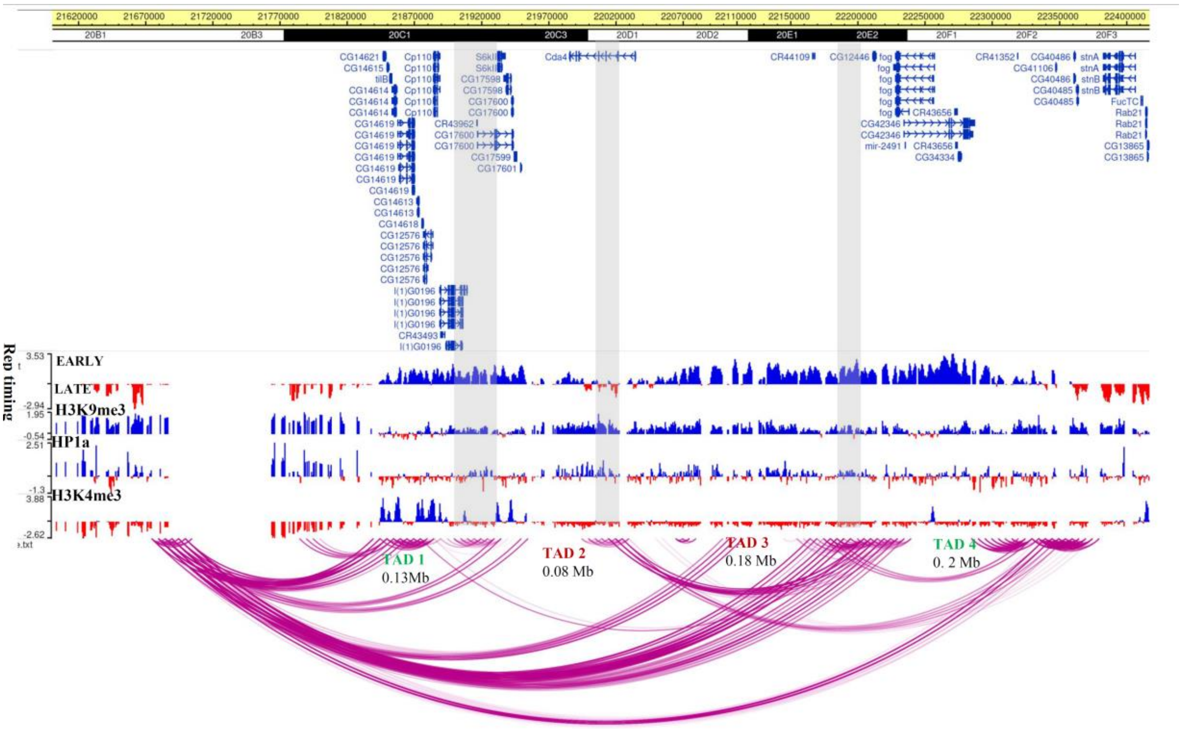

Fig S10: **Overlaps of Het TADs with the replication timing domains in the pericentromeric regions-** Active Het TADs are generally enriched for early replicating regions. Overlay of the replication timing data with long-range interactions in **c) c**hrX. Grey bars indicate TAD borders.

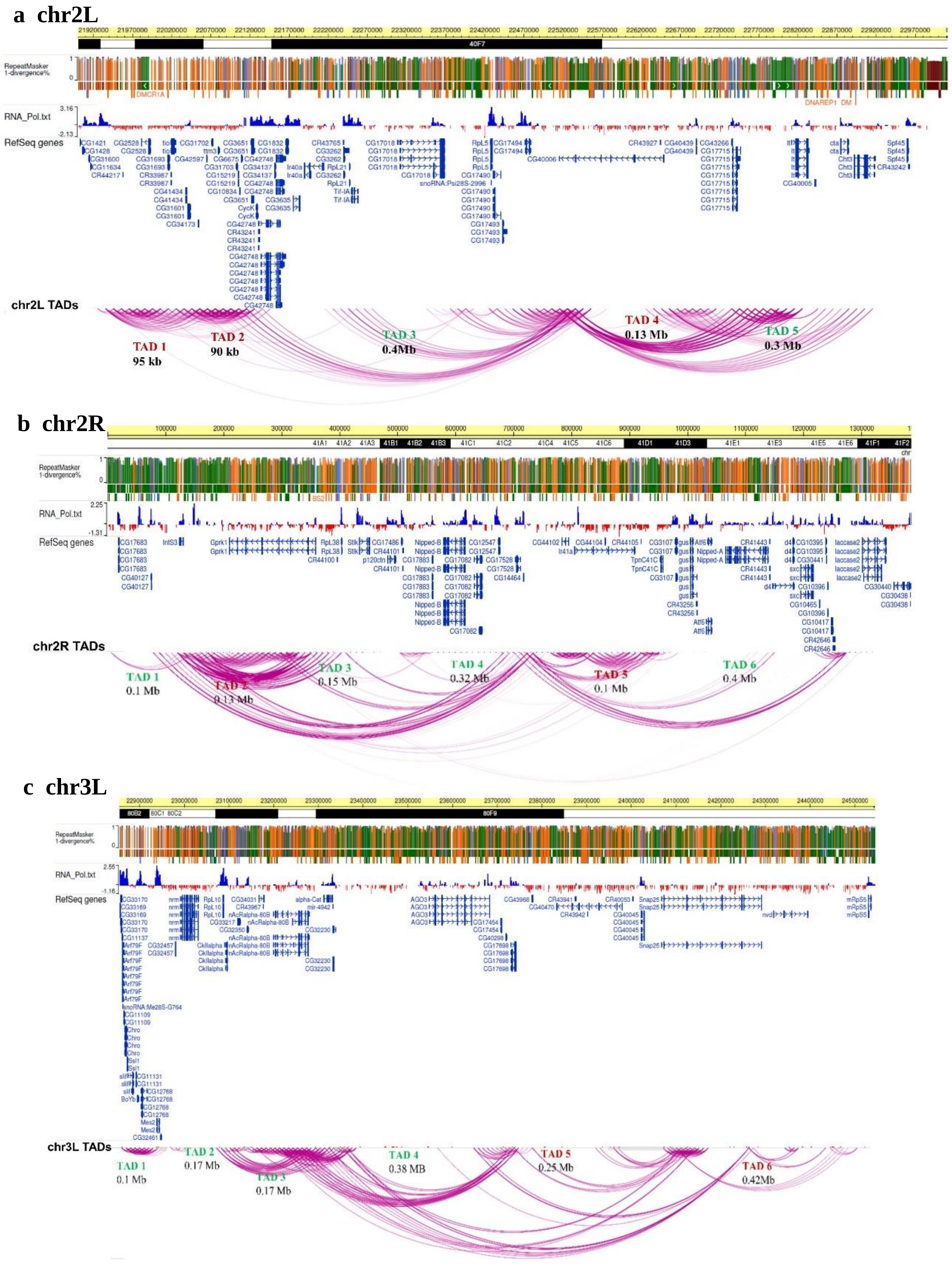

Fig S11: **Overlaps of Het TADs with the RNA Pol II ChIP peaks in the pericentromeric regions- a)** chr2L**, b)** chr2R, **c)** chr3L

d chrX

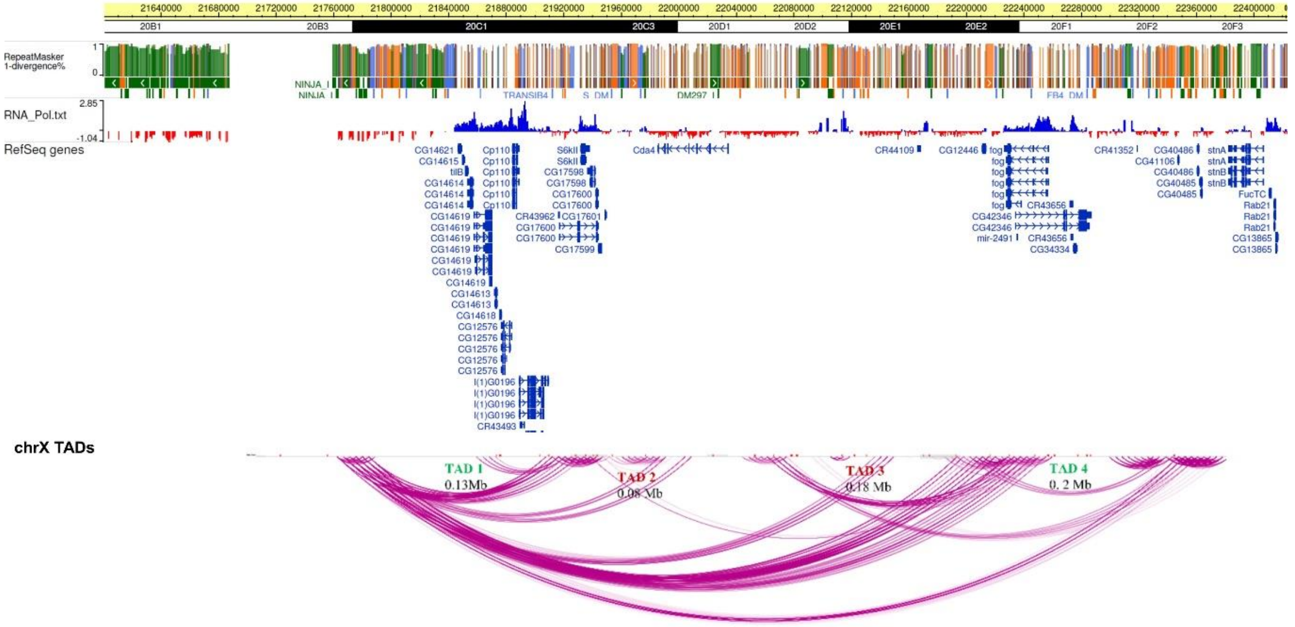

Fig S11: **Overlaps of Het TADs with the RNA Pol II ChIP peaks in the pericentromeric regions- d)** Chr X

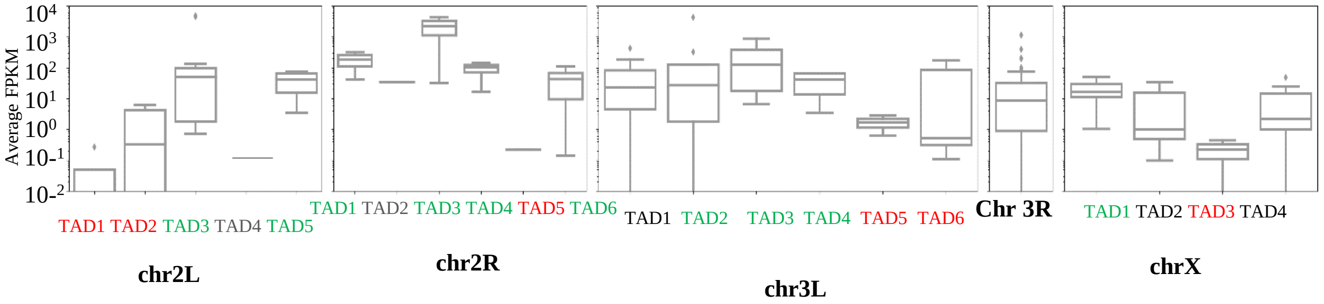

Fig S12: Correlation of Het TAD with expression levels of heterochromatic genes- The expression values of the genes within each TAD on each chromosome plotted as boxplots of average FPKM values. Narrow range of gene expression within a TAD indicate TADs with similarly expressing genes whereas the broad range indicate the TAD encompasses genes with varying expression levels. TADs with most genes with FPKM values < 10^0 are inactive. Inactive TADs are marked in red and active in green. Few TADs have genes with wide range of expression levels marked in black (chr3L TAD1, chrX TAD 2 and 4) or genes largely into spanning adjacent TADs (*CG40046* in chr2L TAD4 and *Gprk1*in chr2R TAD 2). chr3R has genes of varying expression levels.

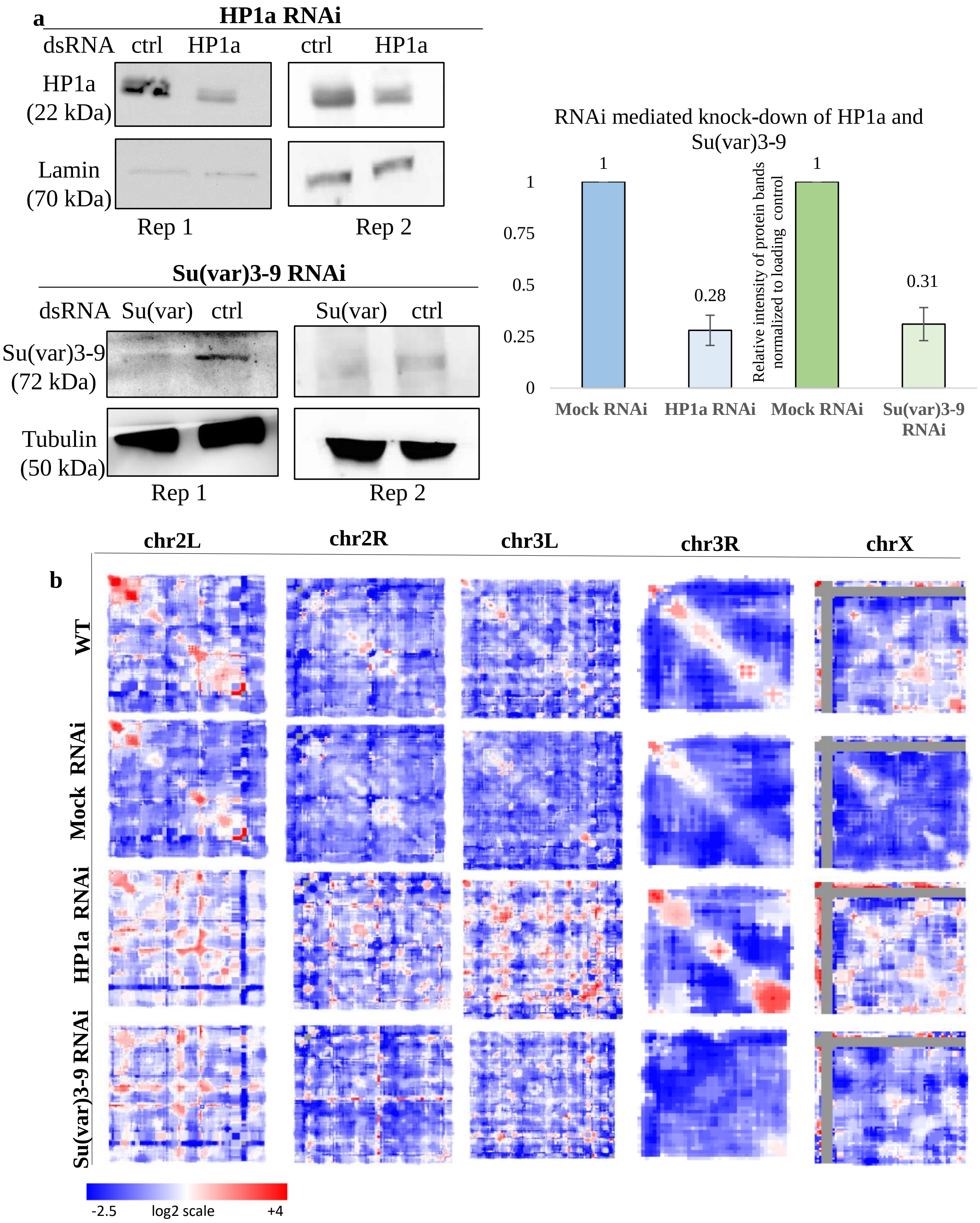

Fig S13: RNAi mediated depletion of HP1a and Su(var)3-9 and effect on genome organization- a) Immunoblot showing the knock-down of HP1a and Su(var)3-9 in S2 cells (n=2) using dsRNA mediated RNA interference and quantitative analysis. **b)** Heatmap for 5 chromosome arms in WT, Mock GFP RNAi, HP1a RNAi and Su(var)3-9 RNAi treated S2 cells. There is an increase in longer range DNA interactions across the diagonal and also on either side of it, indicating inter TAD interactions.

a

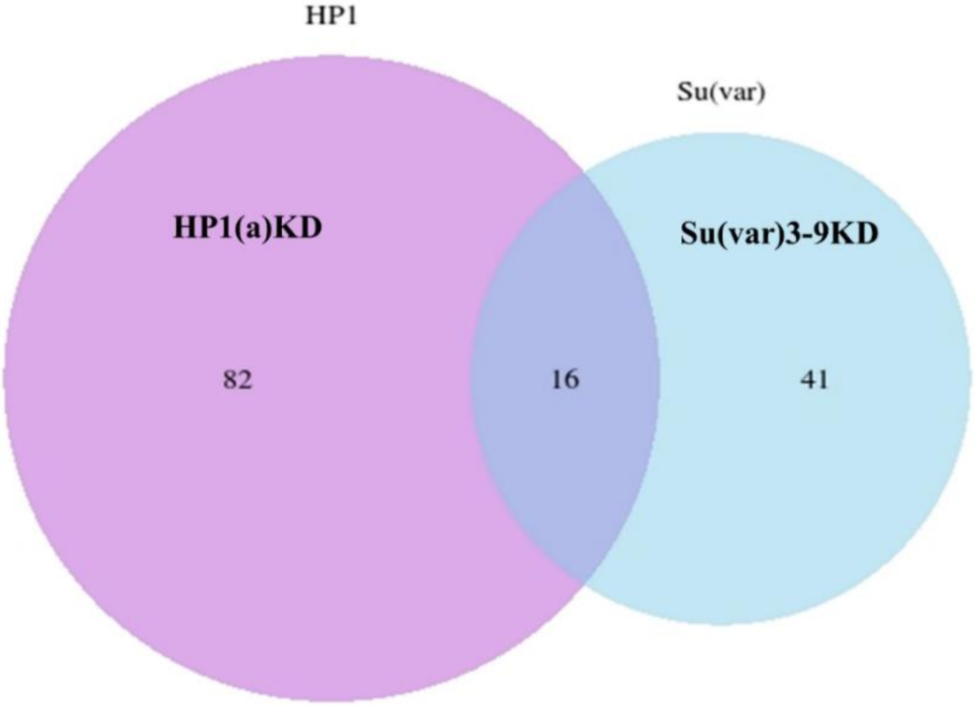

b

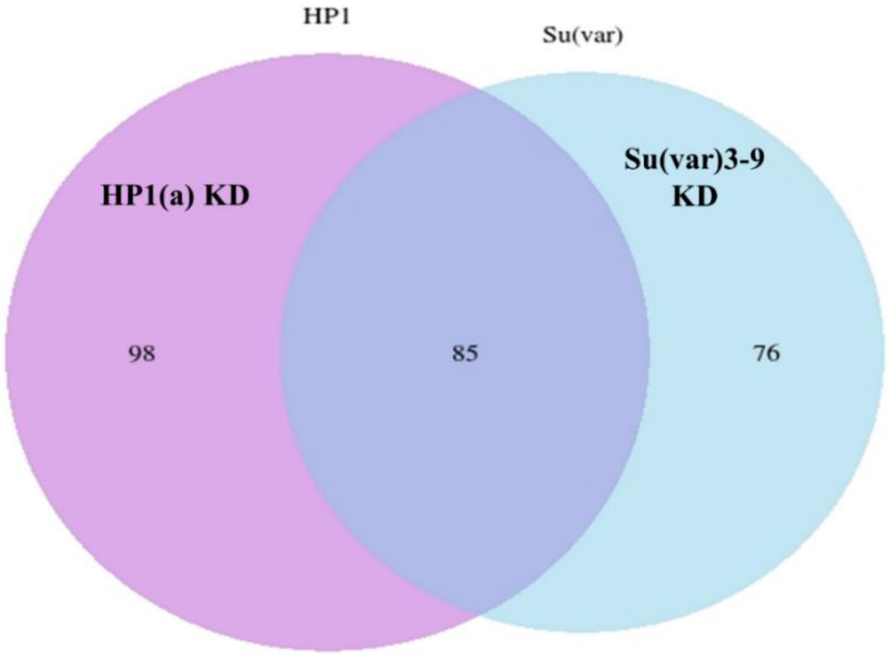

Fig S14: Effects of RNAi mediated depletion of HP1a or Su(var)3-9 on genome-wide transcriptome - Venn diagram showing the number of genes **a)** upregulated and **b)** down-regulated in HP1a and Su(var)3-9 knockdown conditions and the common between the two sets across the entire S2 cell transcriptome.

Table 1: List of TAD and TAD boundary coordinates

TADs

Chr	start	end	size (bp)
chr2L	21900975	21996057	95082
chr2L	22026057	22116057	90000
chr2L	22136057	22536057	400000
chr2L	22556057	22686057	130000
chr2L	22706057	23011544	305487
chr2R	1	109746	109754
chr2R	129746	259746	130000
chr2R	299746	449746	150000
chr2R	469746	789746	320000
chr2R	799746	899746	100000
chr2R	909776	1385689	475913
chr3L	22855576	22956370	100794
chr3L	22976370	23146370	170000
chr3L	23166370	23336370	170000
chr3L	23356370	23736370	380000
chr3L	23756370	24006370	250000
chr3L	24116370	24543557	427187
chrX	21600796	21779812	(seq gap)
chrX	21784812	21919812	135000
chrX	21924812	22004812	80000
chrX	22019812	22199812	180000
chrX	22209812	22422827	213015

TAD boundaries

Chr	start	end	size (bp)
chr2L	21996057	22016057	30000
chr2L	22116057	22136057	20000
chr2L	22536057	22556057	20000
chr2L	22686057	22706057	20000
chr2R	109746	129746	20000
chr2R	259746	299746	40000
chr2R	449746	469746	20000
chr2R	789746	799746	10000
chr2R	889746	909776	10000
chr3L	22956370	22976370	20000
chr3L	23146370	23166370	20000
chr3L	23336370	23356370	20000
chr3L	23736370	23756370	20000
chr3L	24006370	24116370	110000
chrX	21779812	21789812	
chrX	21914812	21924812	10000
chrX	22004812	22019812	10000
chrX	22199812	22209812	15000

Genes falling in TAD borders (low expression genes in grey)
2L*: CG31693, CG3651, CG15218, CG40006, CG40439*
2R: CG17665, CG40129, CG40293, CG41440, CG17704, CG33492
3L*:CG32230, CG17698, CG40045, CG40452*
X:CG17600 CG32499

Table 2a: List of differentially expressed genes in HP1a or Su(var)3-9 RNAi conditions (p value < 0.05; Student two-sided T-test)

**HP1a RNAi**	**Su(var)3-9 RNAi**
CG11739	CG14636
stnB	CG17159
stnA	CG34357
CG12581	CG40006
CG14615	CG41434
l(1)G0196	CG6675
Usp2	CG9780
CG14636	CR33294
CG17514	CR41501
Dsk	
Cht3	
CG31525	
Ir41a	
CG40006	
CG40198	
MFS17	
Fog	
CG9780	
CR41501	
18SrRNA-Psi:CR41602	

Table 2b: Average FPKM values of H3K9 methyltransferases in the RNAi conditions

**Gene**	**WT (S2 cells)**	**HP1a RNAi**	**Su(var)RNAi**
**G9a**	6.24	7	7
**Setdb1/egg**	25.43	31	29

Table 3: List of primers

**Primers for validating 5C interactions by 3C-PCR**	
Primer name (chr –5Cprimer number) primer pairs	Sequence (5'-3')
2L For8_ PP1	CCTGCCATCAATAGTCCGAAA
2L For99_ PP1	AGCCGTATCGTCCGTAAG
2L For 113 _PP2	CAGGTAACCATGAAAGCAAAGTAA
2L For209_ PP2	GCTTAACAGCTCTCGCTCAT
2R For139 _PP3	CCGACTTCCCACAGAACAAA
2R For251_ PP3	GAGCACGAAAGCATCAGAATTG
2R For259_PP4	CCTTCGGAATGTCATCCTCATT
2R For270_PP4	GCCAGAAGAGAGTACTGACAAA
2R For280_PP5	GGCTGACACCAGTCAAGAAA
2R For312_PP5	CGTCTCCCACAAGAAGCTTAAA
2R For317_PP6	TATCGACCATACTGTCGAACTTT
2R For423_PP6	TACTTTGAGGGCACGCTTT
3L For5_PP7	GAGTGACAAGAAGCGTCTGC
3L For36_PP7	CACTCACCTCACGCAAT
3L For61_PP8	GAGGAACATACCCGGCATAC
3L For164_PP8	ACGACTTCTGGTAGCTCTCTAA
3R For27_PP9	ACTCCCAAGTGCACAGTAATC
3R For62_PP9	CCTTGTGCTGGTCACACTTAT
X For67_PP10	TTGTACTCCTCGATGAAGCTG
X For85_PP10	GCCTTAATTGTCCGCAGATTG
**Primers for RNAi mediated Knockdown**	
EGFP_F	taatacgactcactatagggaCTACGGCGTGCAGTGCTTCA
EGFP_R	taatacgactcactatagggaCTACGGCGTGCAGTGCTTCA
HP1.F1	taatacgactcactatagggaGCCCTCTGGCAATAAATCAA
HP1.R1	taatacgactcactatagggaCAGGATAGGCGCTCTTCGTA
HP1.F2	taatacgactcactatagggaTGGAGTACTATCTGAAATGGAAGG
HP1.R2	taatacgactcactatagggaCGCCTCGTACTGCTGGATA
Su(var)3-9_F1	taatacgactcactatagggaTATTGAATGCGTCGAGATGG
Su(var)3-9_R1	taatacgactcactatagggaAGAGGTTCTCGTAGGGCACA
ADD1 F1	taatacgactcactatagggaACCCCAACGTAGATCTGGTG
ADD1 F2	taatacgactcactatagggaTGCTTGCGAGTATCTTGTGG
ADD1 R1	taatacgactcactatagggaCAGAAGACGTAGGGGCAAGT
ADD1 R2	taatacgactcactatagggaGCTCCATCTCGCGTAGGTAA
**Primers for quantitativePCR**	
ADD1_RT_fwd	ACCCCAACGTAGATCTGGTG
ADD1_RT_rev	CAGAAGACGTAGGGGCAAGT
nipped A fwd	CAGCGGTCAAGTTGTTGAAC
nippedA rvs	GACGGATATGCCGGACTTTG
light fwd	GGTCACAATTTCAGGGTGCT
light rvs	AGAAGCGTCCGAAGCACTTA

RpL15 fwd	GACCTACTCGCCCGGATAA
RpLl5 rvs	TACACCATGACTCTTCG
DIP1_fwd	GATCATCATCCGGTGCATCG
DIP_rvs	GATTCACGGCCGTTGTCAC
Cht3_fwd	CGTGGGAGCATTTAGTTGGT
Cht3 _rvs	ATGTACAGCCACCCAGAAGG
CG40006 Fwd	CTGTACTGCCCTTGTGCTGA
CG40006 rvs	TGTGACTGAAGCGGCTAATG
Intergenic_fwd	AATTGCATCGCAACACAATGAG
Intergenic_rvs	TCGTGAAATGTTTGCTACTGGAATA
dATF2_fwd	CCCAGTAGCGCCAGTCTGA
dATF2_rvs	GTGGATAATGGCCTTCAATCG
Ide_-fwd	CTGAGCGGGGAAATCAATGCG
Ide_rvs	TTGCTGTAGGCATGGTCAGG
CG5094_fwd	CCGAGCACCTAGAAGTGGAT
CG5094_rvs	GCAATTTGCACGGTCATTTA
CG15811_fwd	TGCCCAGGCTTATTGTCTTC
CG15811_rvs	TTCGAGAGACTGCCCAAATC
Rp49_fwd	AAGAAGCGCACCAAGCACTTCATC
Rp49_rvs	TCTGTTGTCGATACCCTTGGGCTT
**3C controls**	
1K	AAGCCGCAGGAGTTTCTAAC
2K	CACGGGAAAAACTACTGAAAG

Table 4: List of antibodies

**Antibody against**	**Source**	**Dilutions used**
HP1a	DSHB (C1A9)	1:1000
Su(var)3-9	Abcam ab4811	1:1000
Lamin	In-house (dm0)	1:2000
Tubulin	Abcam ab6046	1:1000
H3K36me3	Abcam ab9050	2-3µg per ChIP reaction

Table 5: List of Next Generation Sequencing datasets of S2 cell line used from NCBI GEO database or modENCODE

**Genomic Feature**	**Experimental technique**	**Source**
H3K9me2	ChIP seq	modENCODE_3953
H3K9me3	ChIP Chip	modENCODE_313
H3K9ac	ChIP Chip	modENCODE_3765
H3K27ac	ChIP Chip	modENCODE_3757
H3K4me1	ChIP Chip	modENCODE_3760
H3K4me3	ChIP Chip	modENCODE_3761
H3K36me3	ChIP seq	modENCODE_4715
HP1a	ChIP Chip	modENCODE_3777
HP1b	ChIP Chip	modENCODE_3020
HP1c	ChIP Chip	modENCODE_3291
mod(mdg4)	ChIP Chip	modENCODE_3789
Su(Hw)	ChIP Chip	modENCODE_330
CP190	ChIP Chip	modENCODE_3748
GAF	ChIP Chip	modENCODE_3753
BEAF32	ChIP on chip	modENCODE_3745
CTCF	ChIP seq	modENCODE_2638
dMES4	ChIP seq	GSE56101
dADD1	ChIP seq	GSE56101
Enhancers	STARR-Seq	GSE40739
Boundary Elements	Bioinformatics prediction	cdBEST (http://e-portal.ccmb.res.in/e-space/rakeshmishra/cdBEST.html)
dMES4 KD	RNA seq	GSM1376614/15
RNA Pol II	ChIP seq	modENCODE_329
